# A feed forward loop enforces YAP/TAZ signaling during tumorigenesis

**DOI:** 10.1038/s41467-018-05939-2

**Published:** 2018-08-29

**Authors:** Mandeep K. Gill, Tania Christova, Ying Y. Zhang, Alex Gregorieff, Liang Zhang, Masahiro Narimatsu, Siyuan Song, Shawn Xiong, Amber L. Couzens, Jiefei Tong, Jonathan R. Krieger, Michael F. Moran, Alexandre R. Zlotta, Theodorus H. van der Kwast, Anne-Claude Gingras, Frank Sicheri, Jeffrey L. Wrana, Liliana Attisano

**Affiliations:** 1 Department of Biochemistry, University of Toronto, Toronto, ON M5S 1A8 Canada; 2 Donnelly Centre, University of Toronto, Toronto, ON M5S 3E1 Canada; 3 Department of Molecular Genetics, University of Toronto, Toronto, ON M5S 1A8 Canada; 4 Centre for Systems Biology, Lunenfeld-Tanenbaum Research Institute, Mount Sinai Hospital, Toronto, ON M5G 1X5 Canada; 5 Program in Cell Biology, Hospital for Sick Children, Toronto, ON M5G 0A4 Canada; 6 SPARC BioCentre, Hospital for Sick Children, Toronto, ON M5G 0A4 Canada; 7 Department of Surgery, Division of Urology, University of Toronto, Mount Sinai Hospital and University Health Network, Toronto, M5G 1X5 ON Canada; 8 Department of Pathology, Toronto General Hospital, University Health Network, Toronto, ON M5G 2C4 Canada; 9 Department of Pathology, McGill University and Research Institute of the McGill University Health Center, Montreal, H4A 3J1 QC Canada; 10 Present Address: Department of Biomedical Sciences, College of Veterinary Medicine and Life Sciences, City University of Hong Kong, 999077 Hong Kong, China; 11 City University of Hong Kong Shenzhen Research Institute, Shenzhen, Guangdong 518057 China

## Abstract

In most solid tumors, the Hippo pathway is inactivated through poorly understood mechanisms that result in the activation of the transcriptional regulators, YAP and TAZ. Here, we identify NUAK2 as a YAP/TAZ activator that directly inhibits LATS-mediated phosphorylation of YAP/TAZ and show that NUAK2 induction by YAP/TAZ and AP-1 is required for robust YAP/TAZ signaling. Pharmacological inhibition or loss of NUAK2 reduces the growth of cultured cancer cells and mammary tumors in mice. Moreover, in human patient samples, we show that NUAK2 expression is elevated in aggressive, high-grade bladder cancer and strongly correlates with a YAP/TAZ gene signature. These findings identify a positive feed forward loop in the Hippo pathway that establishes a key role for NUAK2 in enforcing the tumor-promoting activities of YAP/TAZ. Our results thus introduce a new opportunity for cancer therapeutics by delineating NUAK2 as a potential target for re-engaging the Hippo pathway.

## Abstract (web summary)

The Hippo pathway is frequently dysregulated in cancer. Here, the authors identify NUAK2 as negative regulator of the Hippo pathway from a siRNA kinome screen and show that NUAK2 promotes YAP/TAZ nuclear localisation while NUAK2 is a transcriptional target of YAP/TAZ, thus providing a feed forward loop to promote tumorigenesis.

## Introduction

The Hippo signaling pathway plays a central role in regulating cell proliferation, cell fate, and tissue size^1–3^. Accordingly, the pathway has emerged as a tumor suppressive pathway that acts to control the transcriptional activity of two related proteins, YAP (Yes-associated protein) and WWTR1, also referred to as TAZ^4,5^. YAP and TAZ activity is fundamental not only for normal organ growth and many aspects of tissue regeneration but also underlies several key hallmarks of cancer. For example, YAP/TAZ promote acquisition of cancer stem cell (CSC) characteristics, tumor initiation, progression, and metastasis^4–6^.

Unlike traditional signaling pathways, activation of the Hippo pathway can be triggered by a variety of intrinsic or extrinsic cues such as cell contact, polarity, cytoskeletal remodeling, metabolic and nutrient status, or activation of G-protein-coupled receptors^7–9^. Activation of the pathway results in the engagement of a core kinase cassette, and in vertebrates, this cassette is comprised of the sterile20-like kinases, MST1 and MST2 (*hippo* in Drosophila), the Dbf-2-related (NDR) family kinases, LATS1 and LATS2, and the scaffolding proteins Salvador1 (SAV1) and MOB1A/B (Mps one binder 1)^1–3^. Sequential phosphorylation and activation of MST1/2, and then LATS1/2, culminates in the phosphorylation, cytoplasmic sequestration, and then degradation of the LATS-targeted proteins, YAP and TAZ. When the pathway is inactive, YAP/TAZ accumulate in the nucleus, associate with DNA-binding proteins, most notably TEADs, and also with others such as SMADs, RUNXs, p63/p73, and AP-1, and thus drive a pro-oncogenic transcriptional program^1,3,10,11^. Analysis of genome-wide chromatin occupancy has shown that YAP/TAZ-responsive elements are frequently located at long distances from the start of transcription and many of these enhancers are also bound by AP-1^10,11^. Of note, cooperative interactions between AP-1 and YAP/TAZ are important for regulating the expression of genes that drive cell migration and oncogenic growth^10,11^. In line with this tumor-promoting activity, a broad range of aggressive human solid cancers including breast and bladder cancers display widespread activation of YAP and TAZ^4,5,12^. For instance, in breast cancer, TAZ or YAP levels positively correlate with tumor grade, metastasis, and induction of CSC-like activity^13,14^. In bladder cancer patients, YAP or TAZ overexpression is associated with poor prognosis^15,16^. Moreover, YAP/TAZ are thought to confer resistance to targeted therapies in diverse tumors^16^. Thus, there is a compelling case for targeting YAP and TAZ for therapeutic intervention^5,17^.

The molecular pathways whereby upstream signals such as cell polarity, mechanotransduction, energy stress, and hormones control the activity of components of the core kinase cassette are under intense investigation^1–3,7–9,18^. In this regard, several studies have highlighted the contribution of regulatory kinases in this pathway such as mitogen-activated protein kinase kinase kinase kinase (MAP4Ks) that function redundantly with MSTs^19^, and AMP-activated protein kinase (AMPK) family members such as AMPK and microtubule-associated protein/microtubule-affinity regulating kinases (MARKs) that can either enhance or inhibit MST/LATS activity^20–25^ or SIK2, which in Drosophila, inhibits the hippo kinase cassette^26^.

Here, we sought to identify regulatory pathways that promote YAP/TAZ activity in cancer. Using a small interfering RNA (siRNA) kinome screen to monitor YAP/TAZ localization in breast cancer cells, we identified NUAK2, an AMPK family member, as a positive regulator of YAP/TAZ activity that directly inhibits LATS-mediated phosphorylation of YAP/TAZ. Moreover, we uncovered a striking role for NUAK2 as a YAP/TAZ/AP-1 target gene that is critical for robust YAP/TAZ signaling. Accordingly, knocking out *NUAK2* with CRISPR, blocking expression with RNA interference (RNAi) or pharmacological inhibition of NUAK2 activity drives cytoplasmic localization of YAP/TAZ, inhibits YAP/TAZ transcriptional activity, attenuates the growth of diverse cancer cell lines in culture, and decreases tumor growth in an orthotopic breast cancer mouse model. In addition, we show that in human patient samples, NUAK2 expression is elevated in aggressive, high-grade (HG) bladder cancers and strongly correlates with a YAP/TAZ gene signature. Altogether, our studies identify a positive feed forward loop in the Hippo pathway and demonstrate a key role for NUAK2 in promoting YAP/TAZ oncogenic activity.

## Results

### Identification of NUAK2 as positive regulator of YAP/TAZ activity

**Fig. 1:**
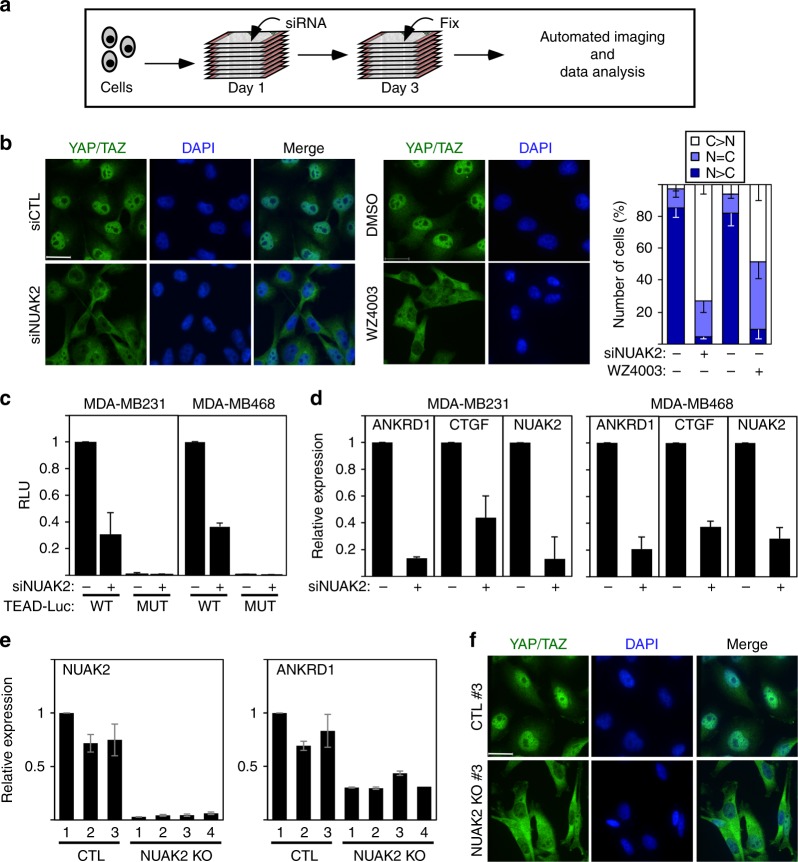
NUAK2 regulates YAP/TAZ localization and transcriptional activity. **a** A schematic depicting the siRNA kinome screen. **b** Blocking NUAK2 with siRNA or WZ4003 (10 μM) for 16 h promotes cytoplasmic localization of YAP/TAZ in MDA-MB231 cells. Quantitated results are plotted as the mean ±  SD (*n* = 3). Scale bars, 25 μm. N nuclear, C cytoplasmic. **c**, **d** Loss of NUAK2 suppresses TEAD-luciferase reporter activity (**c**) and YAP/TAZ target genes expression (**d**) in the indicated breast cancer cell lines. Data are plotted as the mean ± SD (*n* = 3). **e** *NUAK2*KO in MDA-MB231 cells decreases expression of the YAP/TAZ target gene, ANKRD1. Data are plotted as the mean ±  the range of two experiments. **f** *NUAK2* KO in MDA-MB231 cells promotes cytoplasmic localization of YAP/TAZ. Representative images from one clone are shown

To uncover cancer-relevant activators of YAP/TAZ, we conducted a siRNA kinome screen in MDA-MB231 breast cancer cells using an imaging-based YAP/TAZ subcellular localization assay as readout (Fig. 1a). This identified NUAK2, a poorly studied kinase^27^, as a gene whose siRNA-mediated loss promoted cytoplasmic localization of YAP/TAZ (Fig. 1b). Accordingly, siNUAK2 blocked YAP/TAZ transcriptional activity as assessed using a TEAD-luciferase reporter and by measuring expression of the endogenous target genes, ANKRD1 and CTGF, in two breast cancer lines (Fig. 1c, d and Supplementary Fig. 1a). MDA-MB231 clones with CRISPR/Cas9-mediated knockout (KO) of *NUAK2* similarly displayed a reduction in transcriptional output and a corresponding decrease in nuclear YAP/TAZ localization (Fig. 1e, f). Consistently, overexpression of NUAK2 increased the activity of the wild-type (WT) but not the mutant TEAD reporter (Supplementary Fig. 1c). In contrast, loss of expression of the closely related NUAK1 did not significantly effect ANKRD1 expression in either parental or *NUAK2* KO cell lines (Supplementary Fig. 1d, e). In rescue experiments of either siNUAK2 or using *NUAK2* KO clones, overexpression of full-length WT or kinase-domain-containing deletion constructs, but not kinase-deficient (K81R) NUAK2, restored YAP/TAZ nuclear levels (Supplementary Fig. 2a, b). Thus, kinase activity is required for NUAK2 function in modulating YAP. To further bolster our conclusions, we tested the effects of two NUAK1/2 kinase inhibitors, the highly specific WZ4003^28^ and a less specific but more potent compound, ON123300^7^. Treatment of MDA-MB231 cells with WZ4003 or ON123300 drove YAP/TAZ into the cytoplasm and inhibited the activity of the TEAD-luciferase reporter (Fig. 1b and Supplementary Figs. 1c and 2c). Thus, NUAK2 functions in a kinase-dependent manner to promote nuclear YAP/TAZ localization and activity.

### NUAK2 functions upstream of LATS

**Fig. 2:**
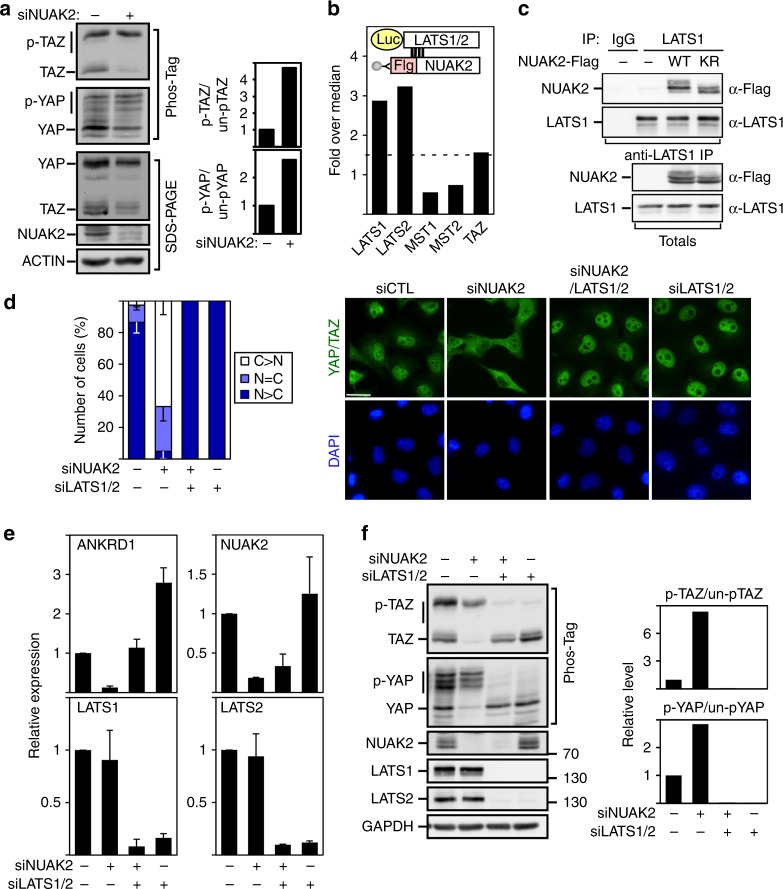
NUAK2 regulates YAP/TAZ activity through LATS. **a** Loss of NUAK2 enhances YAP/TAZ phosphorylation in MDA-MB231 cells. Quantitation of relative phosphorylation levels from blots is shown. **b** NUAK2 interacts with LATS1 and LATS2, but not MST1, MST2, or TAZ in the LUMIER protein interaction screen. **c** Lysates from HEK293T cells, transfected with NUAK2-Flag, were subjected to immunoprecipitation (IP) using anti-LATS1 antibody for endogenous LATS1 and co-immunoprecipitated NUAK2-Flag was detected by immunoblotting. Protein expression levels were confirmed (Totals). WT wild type, KR K81R, kinase dead. **d**–**f** NUAK2 requires LATS to regulate YAP/TAZ localization, phosphorylation, and target gene expression in MDA-MB231 cells. **d** YAP/TAZ localization was quantitated and plotted as the mean ± SD (*n* = 3) with representative images shown on the right. Scale bars, 25 μm. N: nuclear, C: cytoplasmic. **e** Target gene expression was determined by qPCR. Data are plotted as the mean ± SD (*n* = 3). **f** YAP/TAZ phosphorylation was monitored using Phos-Tag gels. Relative phosphorylation levels from blots is quantitated (right)

Phosphorylation of YAP/TAZ by LATS1/2, the downstream kinase in the Hippo core kinase cassette, promotes cytoplasmic accumulation and subsequent degradation of YAP/TAZ^29,30^. Blocking NUAK2 expression or kinase activity using siRNAs or WZ4003, respectively, or analyzing MDA-MB231 *NUAK2* KO clones, revealed an increase in the relative level of phosphorylated versus unphosphorylated YAP and TAZ as assessed on Phos-Tag gels or using phospho-specific antibodies that recognize the LATS-targeted Ser127 site in YAP while siNUAK1 had no effect (Fig. 2a and Supplementary Figs. 1a, b, d, and 3a, b). The expected reduction in total YAP/TAZ levels that occurs subsequent to phosphorylation^29,30^ was also evident (Fig. 2a). Concordantly, induced overexpression of NUAK2 in MDA-MB231 stables reduced the levels of phosphorylated YAP/TAZ (Supplementary Fig. 3c).

These findings, together with our observation of a predominantly cytoplasmic localization for NUAK2 (Supplementary Fig. 3d), suggested that NUAK2 might act directly on the Hippo cassette. Mining of our previous LUMIER protein interaction map^31,32^ revealed that NUAK2 interacted with LATS1 and LATS2, but not MST1, MST2, or TAZ (Fig. 2b). We confirmed the interaction of endogenous LATS with Flag-tagged WT NUAK2 (Fig. 2c) and determined that the kinase domains (KDs) of both NUAK2 and LATS1 are sufficient for association (Supplementary Fig. 3e,f). We next examined whether LATS is required for NUAK2 function on YAP/TAZ. As expected, loss of LATS1/2 promoted nuclear localization of YAP/TAZ, decreased YAP/TAZ phosphorylation, and enhanced expression of YAP/TAZ target genes (Fig. 2d–f). Notably, loss of LATS1/2 also reversed the inhibition of YAP/TAZ nuclear accumulation, target gene expression, and induction of YAP/TAZ phosphorylation caused by siNUAK2 (Fig. 2d–f). LATS phosphorylates YAP on five serine residues^29^. Live cell imaging of MDA-MB231 cells stably expressing WT Clover-YAP (Supplementary Movie 1 and Supplementary Fig. 4a, b) showed rapid relocalization of YAP upon WZ4003 addition (<30 min). However, YAP mutated in all five LATS-targeted sites (Clover-YAP 5SA) remained nuclear (Supplementary Movie 2 and Supplementary Fig. 4a, b).

### NUAK2 phosphorylates and inhibits LATS activity

**Fig. 3:**
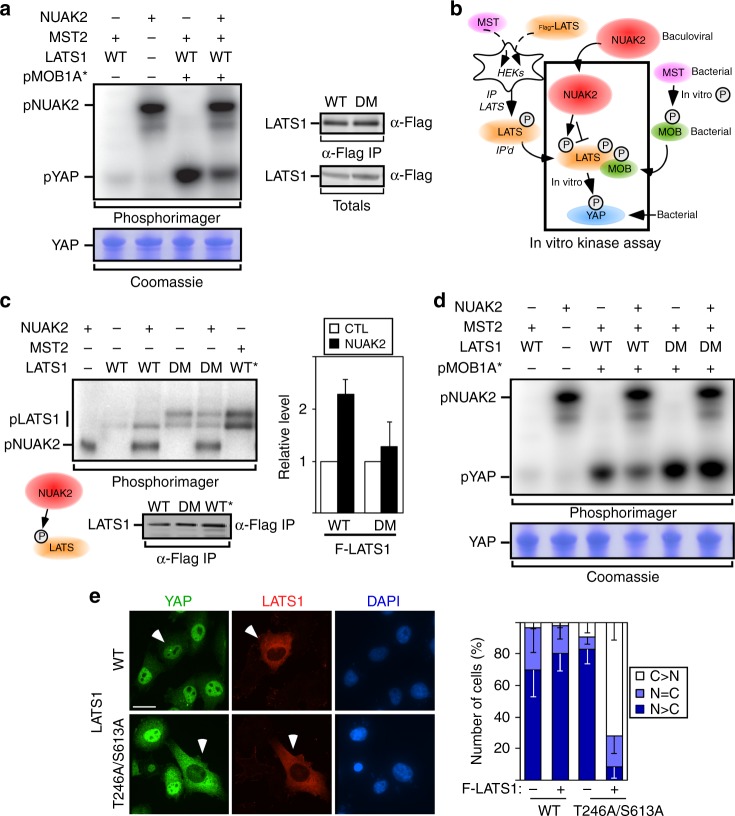
NUAK2 phosphorylates and inhibits LATS activity. **a** Purified NUAK2 blocks LATS-mediated phosphorylation of YAP in an in vitro kinase assay. WT wild type, pMOB1A* the modified active variant (T12 to T353) of MOB1A (see Methods). Levels of immunoprecipitated LATS used for **a** and **d** are shown (right). **b** A schematic depicting in vitro kinase assay. The method used to produce and activate each component is indicated. **c** NUAK2 phosphorylates the wild-type (WT) but not the T246/S613 double mutant (DM) LATS in an in vitro kinase assay. Levels of immunoprecipitated WT and DM LATS are comparable. Relative phosphorylation levels from blots is quantitated and plotted as the mean ± SD (*n* = 5) (right). WT* MST-activated WT LATS. Note that the DM mutant migrates as a doublet, similar to MST-activated WT LATS, consistent with the notion that endogenous NUAKs have a reduced ability to block LATS1 activation. **d** NUAK2 does not inhibit the activity of the LATS T246/S613 double mutant (DM) in an in vitro kinase assay. **e** Expression of LATS T246/S613 (DM) induces cytoplasmic localization of YAP in MDA-MB231 cells. Quantitated results are plotted as the mean ± SD (*n* = 3). Scale bar, 25 μm. N: nuclear, C: cytoplasmic

We next sought to evaluate whether NUAK2 alters LATS1 activity in vitro by reconstituting the Hippo kinase cassette. Within this module, MST phosphorylates both LATS and the scaffolding protein MOB1, both of which are required for efficient activation of LATS^33^. Thus to test the effect of NUAK2 in this assay, Flag-LATS1 was immunoprecipitated from HEK293T cells co-expressing MST2 and phosphorylation of a YAP peptide was assayed in the presence of purified, in vitro phosphorylated MOB1^34^ (Fig. 3a, b). We previously showed that introduction of amino acid substitutions into MOB1A facilitates binding to NDR1/LATS kinases^35^. Concordantly, we observed that addition of pMOB1, either WT or the modified active variants (T12 to T353 or T12 to T367) strongly stimulated LATS1 activity towards YAP, and in all cases, LATS activity towards YAP was inhibited by NUAK2 (Supplementary Fig. 5a).

To determine whether NUAK2 might phosphorylate LATS, we performed an exploratory mass spectrometric analysis of immunoprecipitated LATS1 that had been subjected to an in vitro kinase assay in the presence of NUAK2. This approach identified two sites, Thr246 and Ser613, as being phosphorylated by WT but not kinase-deficient NUAK2. Analysis by an in vitro kinase assay revealed that NUAK2 could phosphorylate immunoprecipitated LATS and that this phosphorylation was lost in the LATS double mutant (DM), T246A/S613A (Fig. 3c). This mutant retained catalytic activity and was fully capable of phosphorylating YAP in vitro, but most notably, this activity was no longer blocked by NUAK2 (Fig. 3d). Concordantly, in MDA-MB231 cells, low-level expression of the mutant but not WT LATS induced cytoplasmic YAP/TAZ, consistent with the notion that this mutant variant escapes NUAK2-dependent inhibition (Fig. 3e).

Analysis of the single LATS T246A or Ser613A mutants revealed that both single mutants displayed resistance to inhibition by NUAK2 in the YAP/TAZ localization assay, but this was most prominent in the case of the Ser613A mutant (Supplementary Fig. 5b). Consistently, in the in vitro kinase assay, the extent of loss of NUAK2-mediated phosphorylation of LATS was most prominent when Ser613 was mutated (Supplementary Fig. 5c). We next analyzed LATS phosphorylation in HEK293 cells using mass spectrometry. Immunoprecipitated LATS displayed a 1:2 stoichiometry of phosphorylated to non-phosphorylated Ser613 that was increased upon co-expression of WT but not kinase-deficient NUAK2 (Supplementary Fig. 5d) Moreover, pre-treatment of cells with WZ4003 or ON123300, which inhibit endogenous NUAKs resulted in a decrease in pSer613 levels (Supplementary Fig. 5e). In the case of T246, phosphorylation levels were very low, preventing accurate determination of NUAK-induced changes.

### *NUAK2* is a YAP/TAZ target gene that acts in a feed forward loop

**Fig. 4:**
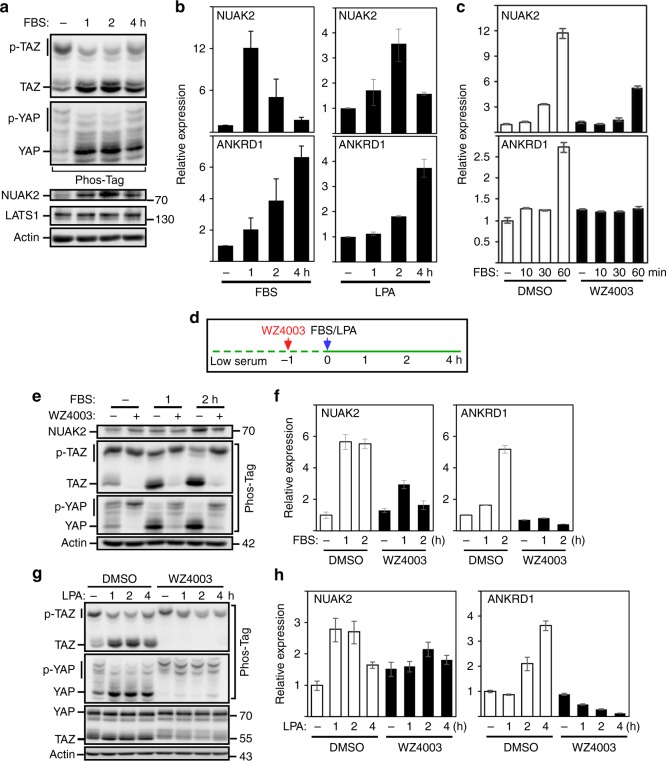
NUAK2 activity is involved in serum-induced or LPA-induced YAP/TAZ dephosphorylation and activation. **a**, **b** FBS and LPA activate YAP/TAZ and induce NUAK2 expression. YAP/TAZ phosphorylation status was monitored using Phos-Tag gels (**a**), while NUAK2 and ANKRD1 mRNA expression was determined by qPCR at the indicated times. Expression data are plotted as the mean ± SD (*n* = 3). **c**–**h** WZ4003 (10 μM) blocks serum/LPA-induced NUAK2 and ANKRD1 mRNA expression. FBS-induced or LPA-induced YAP/TAZ dephosphorylation (**e** and **g**), or NUAK2 and ANKRD1 mRNA expression (**c**, **f**, **h**) was monitored at the indicated times (**d**). Expression data are plotted as the mean ± range for a representative experiment (**c**, **f**, **h**)

**Fig. 5:**
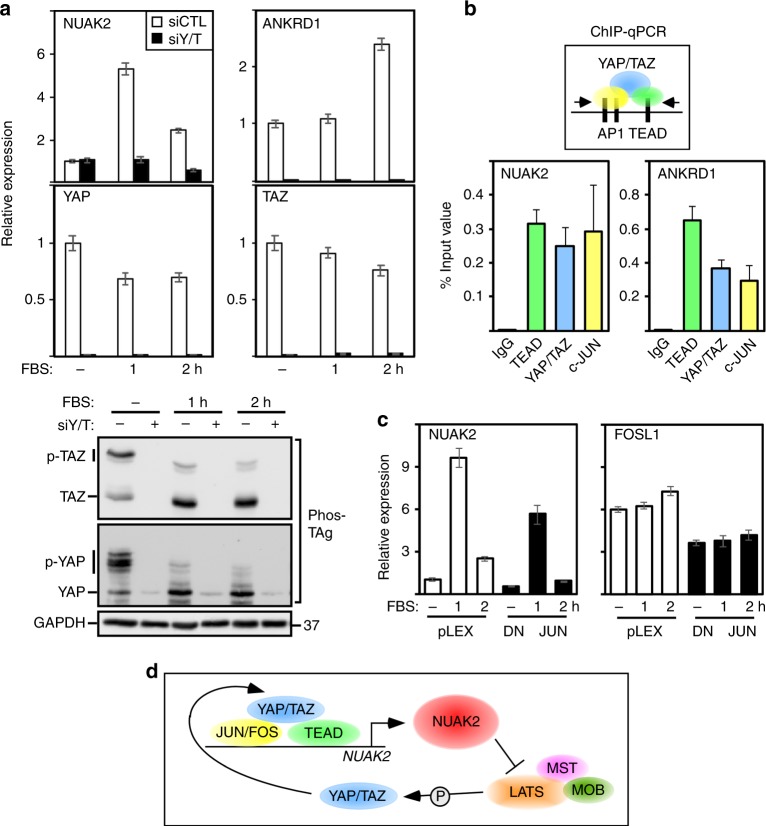
YAP/TAZ and NUAK2 act in a positive feed forward loop in MDA-MB231 cells. **a**, **b** Loss of YAP/TAZ blocks FBS-induced YAP/TAZ dephosphorylation and expression of NUAK2. YAP/TAZ phosphorylation was monitored using Phos-Tag gels (bottom panel) and mRNA expression of the indicated genes (top panel) was determined by qPCR. Expression data are plotted as the mean ± range of a representative experiment. **b** ChIP-qPCR analysis shows that YAP, TEAD, and JUN bind to the *NUAK2* and *ANKRD1* enhancers. Data are plotted as the mean ± SEM (*n* = 4). **c** Dominant-negative JUN (DN-JUN) attenuates FBS-induced NUAK2 expression at levels comparable to the effects on the JUN target gene, FOSL1. Expression data (qPCR) are plotted as the mean ± range of a representative experiment. **d** A model depicting NUAK2 and YAP/TAZ function in a positive feed forward loop

Lysophosphatidic acid (LPA) or serum, which contains LPA, activates G-protein-coupled receptors, which in turn inhibit LATS kinases to promote YAP/TAZ activity^36^. MDA-MB231 cells stimulated with serum or LPA promoted dephosphorylation of YAP/TAZ as expected^36^, but also rapidly induced NUAK2 messenger RNA (mRNA) and protein expression that preceded the increase in ANKRD1 (Fig. 4a, b and Supplementary Fig. 6a). Pre-treatment with WZ4003 for 1 h or as short as 15 min blocked serum-induced and LPA-induced dephosphorylation of YAP/TAZ and induction of ANKRD1 expression (Fig. 3c–h and Supplementary Fig. 6b), suggesting that NUAK2 enhances serum/LPA-induced activation of YAP/TAZ. Unexpectedly, NUAK2 expression was also attenuated by WZ4003 but had no effect on NUAK1 (Fig. 4c, f, h and Supplementary Fig. 6c), suggesting that *NUAK2* might itself be a YAP/TAZ target gene. Indeed, siRNA-mediated knockdown of YAP/TAZ abrogated expression of NUAK2 but had minimal effects on NUAK1 (Fig. 5a and Supplementary Fig. 6d).

Widespread co-occupancy by TEADs and AP-1 transcription factors (i.e., JUN, FOS, and FRA) on distal enhancers of YAP/TAZ target genes has been reported^10,11^, and given that occupation of AP-1 sites is stimulated by serum and LPA^37^, we investigated whether NUAK2 might be cooperatively activated by serum and YAP/TAZ. For this, we conducted chromatin immunoprecipitation (ChIP)/qPCR on an enhancer element identified as binding JUN/FRA, YAP/TAZ, and TEAD in two independent genome-wide ChIP-seq studies^10,11^. Our analysis revealed that YAP/TAZ, TEAD, and JUN were bound to the *NUAK2* enhancer similar to that reported^10^ for the control *ANKRD1* gene element (Fig. 5b). Overexpression of dominant-negative JUN, which can partially block all AP-1 complexes^10,11^, decreased serum-mediated NUAK2 induction to levels comparable to that of the bona fide AP-1 target, FOSL1 (Fig. 5c). Thus, AP-1 proteins and YAP/TAZ drive high-level activation of NUAK2 expression. Altogether, our results provide compelling evidence for a positive feed forward loop in the Hippo pathway in which YAP/TAZ induce expression of NUAK2, which in turn acts to inhibit LATS to enforce a strong YAP/TAZ transcriptional program (Fig. 5d).

### NUAK2 promotes cell and tumor growth in diverse cancer contexts

**Fig. 6:**
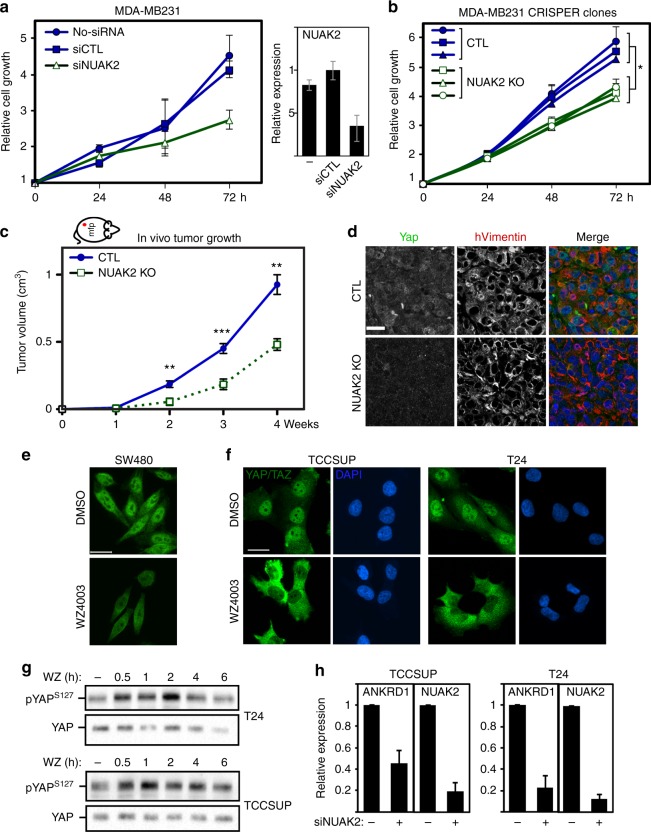
Loss of NUAK2 inhibits in vitro cell and in vivo tumor growth of MDA-MB231 cells. **a**, **b** Loss of NUAK2 expression using siRNA or in *NUAK2* knockout clones inhibits cell growth as measured by the SRB assay. Data are plotted as the mean ± SD of a representative experiment (**a**) or mean ± SEM (*p value <0.05, unpaired two-tailed *t* test) of three to five independent experiments per clone (**b**). NUAK2 knockdown efficiency in the siRNA experiment (**a**) is plotted as the mean ±  range of a representative experiment. **c** Loss of NUAK2 reduces tumor growth in vivo. Data are plotted as the mean ± SD (**p <0.005, ***p <0.001, unpaired two-tailed *t* test). **d** YAP levels are reduced in MDA-MB231-derived *NUAK2*knockout tumor cells, identified by co-staining with human vimentin. **e**–**g** WZ4003 (10 μM) induces cytoplasmic relocalization of YAP/TAZ (**e**, **f**) and YAP phosphorylation (**g**) in the indicated cell lines. **h** Loss of NUAK2 decreases YAP/TAZ target gene expression in bladder cancer cells. Expression data (qPCR) are plotted as the mean ± SD (*n* = 3)

Nuclear YAP/TAZ drives cancer progression^1–4^; thus, we next examined the contribution of NUAK2 to cell growth or tumor formation. MDA-MB231 cells lacking NUAK2 expression either using siRNAs or CRISPR/Cas9-mediated KO displayed a decreased rate of cell growth (Fig. 6a, b). In parallel, analysis of tumor growth in vivo, using an orthotopic mouse mammary model, revealed robust tumor growth between 1 and 4 weeks in controls, whereas *NUAK2* KOs displayed reduced tumor volume increases (Fig. 6c). Staining of sections revealed a dramatic reduction in the levels of YAP/TAZ in the *NUAK2* KO tumor cells (Fig. 6d) consistent with our observations in cell-based assays (Fig. 1f).

*NUAK2* is located within a chromosomal region, 1q32.1, that has been reported to be frequently amplified in human breast cancers^38^; however, in a preliminary analysis (cBioportal), we did not observe a strong correlation between NUAK2 expression and breast cancer grade in a pooled cohort of patients. However, as YAP overexpression is associated with numerous solid cancers^4,5,12^, we next considered whether NUAK2 might also have a role in other contexts. Inhibition of NUAK2 with WZ4003 blocked nuclear accumulation of YAP/TAZ in SW480, an APC mutant colorectal cancer cell line, as well as in TCCSUP, T24, and RT112, three bladder cancer-derived lines (Fig. 6e, f and Supplementary Fig. 7a). Detailed analysis in TCCSUP and T24 cells showed that the effect of WZ4003 was rapid (<30 min), correlated with increased YAP/TAZ phosphorylation, and that ON123300 similarly induced cytoplasmic localization of YAP/TAZ (Fig. 5g and Supplementary Fig. 7b–d). In line with this, abrogation of NUAK2 expression using siRNAs resulted in the expected decrease in YAP/TAZ protein levels as visualized by blotting and immunofluorescence microscopy (Supplementary Fig. 8a, b) and reduced YAP/TAZ target gene expression in all four cell lines (Fig. 6h and Supplementary Fig. 8c). Moreover, as in MDA-MB231 cells (Fig. 4b), treatment of T24 and TCCSUP cells with FBS-induced NUAK2 but not NUAK1 expression (Supplementary Fig. 8d). Thus, NUAK2 modulates YAP/TAZ in diverse cancer contexts. Interestingly, in TCCSUP cells, siNUAK1 also decreased ANKRD1 expression, suggesting that NUAK1 might also contribute to YAP signaling in a cell context-dependent manner, though further experiments are required to more fully address the contribution of NUAK1 (Supplementary Fig. 8e).

### NUAK2 expression is correlated with bladder cancer tumor grade and progression

**Fig. 7:**
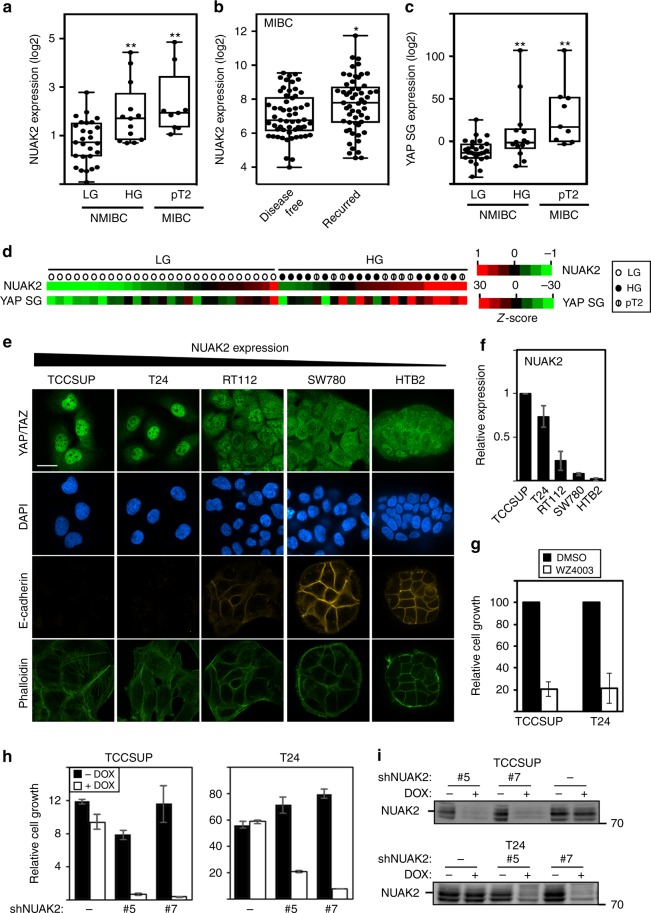
NUAK2 is associated with high-grade human bladder cancer. **a**, **c** Expression of NUAK2 and the YAP Signature Genes (YAP SG) is enhanced both in high-grade non-muscle-invasive (HG-NMIBC; *n* = 13, **p = 0.0071) and muscle-invasive (pT2 MIBC; *n* = 9, **p = 0.0033) bladder cancers^39^ as compared to LG-NMIBC (*n* = 27) using an unpaired *t* test. **b** Elevated expression of NUAK2 is correlated with disease recurrence in a cohort^41^ of MIBC patient samples with *n* = 57 disease free and *n* = 56, recurred samples (*p = 0.0172). Box and whisker plots show the median (line in the box), first and third quartiles (lower and upper ends of the box), and the minimum and maximum values (whiskers in the plot). Dots represent a single patient sample. **d** A heat map depicting expression of NUAK2 and YAP SG in NMIBC LG (open circle), HG (closed circle), and MIBC pT2 (slashed circle) bladder cancer samples^39^. **e** Characterization of high-grade-derived and low-grade-derived bladder cancer cell lines. Scale bar, 25 μm. **f** NUAK2 mRNA expression is elevated in HG-derived (TCCSUP and T24) BC cell lines as compared to LG lines. Data are plotted as the mean ± SD (*n* = 3). **g**–**i** Blocking NUAK2 activity with 10 μM WZ4003 (**g**) or NUAK2 expression in stable clones (#5 or #7) using inducible NUAK2 shRNAs (**h**, **i**) inhibits the growth of HG BC cell lines as measured by SRB (**g**) or DAPI (**h**) staining. Data are plotted as the mean ± SD of a representative experiment, *n* = 3 (for TCC) or mean ± range of two experiments (for T24)

To investigate the relevance to human cancers, we focussed on urothelial bladder cancer, the fourth leading cause of cancer in men, as we had noticed grade-associated alterations in YAP/TAZ target gene expression in our dataset^39^. Median expression of NUAK2, but not NUAK1, was significantly increased both in muscle-invasive (pT2; *p* = 0.0033) and HG non-muscle-invasive (HG-NMI; *p* = 0.0071) samples as compared to low-grade (LG) NMI tumors (Fig. 7a and Supplementary Fig. 9a), as well as in a larger independent collection of NMI tumors^40^ (Supplementary Fig. 9b). Moreover, elevated expression of NUAK2, but not NUAK1, is correlated with disease recurrence in a cohort^41^ of muscle-invasive bladder cancer (MIBC) patients (Fig. 7b and Supplementary Fig. 9c). HG-NMI and MI (pT2) tumor samples also displayed a significant increase in median expression of a YAP 57 gene signature^13^ (Fig. 7c). Importantly, NUAK2 expression was strongly correlated with the overall YAP gene signature (PCC = 0.78, *p* = 0.003) (Fig. 7d and Supplementary Fig. 9d, e), as well as with many YAP target genes, when assessed individually (PCC >0.7; Supplementary Table 1).

To further pursue the association of NUAK2 with tumor grade, we further analyzed cell lines established from HG-MI (TCCSUP and T24; grade 3 or 4), HG-NMI (RT112: grade 2) or LG (SW780 and HTB-2; grade 1) bladder cancer patient tumors^42^. As expected, the LG-derived lines expressed E-cadherin, while HG-derived lines did not (Fig. 7e). Moreover, consistent with results from primary patient tumors, the relative levels of NUAK2 were correlated with grade with the highest expression found in the HG-MI lines, while MST and LATS levels were similar (Fig. 7f and Supplementary Fig. 10a). In line with this, a predominantly nuclear localization for YAP/TAZ was evident in HG-MI TCCSUP and T24 cells versus cytoplasmic for the LG lines, while RT112 cells displayed an intermediate phenotype (Fig. 7e). As described above, WZ4003-induced or siNUAK2-induced cytoplasmic YAP in the HG TCCSUP, T24, and RT112 cells (see Fig. 6f and Supplementary Figs. 7a, b and 8a), but had no effect in LG HTB-2 cells (Supplementary Fig. 10b). Concordantly, treatment of TCCSUP and T24 cells with WZ4003, siNUAK2, or inducing shNUAK2 in multiple stable clones resulted in a potent suppression of cell growth (Fig. 7g–i and Supplementary Fig. 10c). Thus, aggressive HG bladder cancers are characterized by elevated NUAK2 expression and YAP/TAZ activity, and either blocking expression or pharmacologically inhibiting NUAK2 is effective in restoring Hippo pathway activity and attenuating cell growth.

## Discussion

Elevated YAP/TAZ activity, frequently found in solid tumors, can drive oncogenesis; however, mutations in pathway components are rare^4,5^. Here, we identified NUAK2 as a direct inhibitor of the Hippo kinase cassette and present the striking discovery that NUAK2 also functions in a feed forward loop that acts to ensure rapid activation and maintenance of a robust YAP/TAZ transcriptional program. Importantly, we show that pharmacological inhibition or loss of NUAK2 expression blocks YAP/TAZ activity and attenuates cancer cell and tumor growth in mice. Moreover, we demonstrate that NUAK2 expression is elevated in HG-NMI bladder cancer patients, a clinically challenging group^43^, and is associated with poor prognosis in aggressive muscle-invasive bladder cancer patients. Taken together, our work has uncovered a new principle of Hippo pathway control in which NUAK2, a key inhibitor of the Hippo cassette, also acts to enforce the pro-oncogenic activities of YAP/TAZ and thus is an ideal target for therapeutic strategies aimed at re-engaging the Hippo pathway.

The Hippo kinase cassette, comprised minimally of MST, LATS, MOB, and SAV, functions in a poorly understood manner involving autophosphorylation and transphosphorylation events, conformational shifts, and alterations in protein–protein interactions that ultimately activate LATS to phosphorylate YAP/TAZ^1–3^. Our mechanistic analyses of NUAK2 activity on this cassette revealed that NUAK2 interacts with and phosphorylates LATS and prevents LATS-mediated phosphorylation of YAP/TAZ both in vitro and in intact cells. We showed that phosphorylation of Ser613, located upstream of the KD, adjacent to the MOB binding site and to a lesser extent the N-terminal localized T246, are required for NUAK2 to inhibit LATS. However, it is worth noting that our analysis of potential phosphorylation sites was not exhaustive, leaving open the possibility that other target sites, such as Ser464, a reported NUAK1 target site^44^, might also be required in the context of the Hippo pathway. As our current understanding how the Hippo kinase cassette functions at the molecular level is quite poor, further investigations are required to gain detailed insight into how exactly NUAK-mediated phosphorylation of LATS ultimately disrupts LATS activity. Nevertheless, our cell-based epistasis analysis clearly show that the removal of LATS abrogates the ability of NUAK2 to inhibit YAP/TAZ nuclear accumulation and target gene expression, indicating that NUAK2 functions upstream of Hippo kinase cassette to control YAP/TAZ transcriptional activity. As deletion of LATS1/2 potently blocked cell growth, likely due to the known Hippo-independent functions of LATS such as cell cycle regulation^45–47^, we were unable to confirm that the effects of NUAK inhibition on cell proliferation also required LATS. Moreover, whether NUAKs might also control YAP/TAZ activity or tumorigenic processes through Hippo-independent mechanisms or by impacting mechanotransduction, a known modulator of YAP/TAZ activity, remains an open question^18,28,44,48,49^.

Our studies on cultured cancer cell lines and using an orthotopic model of mammary tumorigenesis demonstrated that abrogation of NUAK2 expression or activity blocked YAP/YAZ function and attenuated proliferation in diverse cancer contexts. Interestingly, *NUAK2* resides on chromosome 1q32, a region frequently amplified in tumors including breast cancers^50,51^, suggesting that NUAK2 might have a more widespread role than reported here. Our preliminary analysis of breast cancer patient samples did not reveal an association between NUAK2 expression and grade; however, a more thorough analysis of subtypes in breast or other cancers may uncover such links. It is worth noting that the closely related NUAK1 did not act in a feed forward loop in any of the cell lines we tested, but loss of NUAK1 did attenuate YAP/TAZ target gene expression in TCCSUP but not in MDA-MB231 cells. Thus, the contribution of NUAK1 in other cancers requires further examination. NUAK2 and the related NUAK1 are poorly characterized members of the AMPK-like family that includes the eponymous member, AMPK, an extensively studied regulator of energy balance^27^. Interestingly, unlike NUAKs, AMPK blocks YAP/TAZ function in both a Hippo-dependent and Hippo-independent manner^20,52^. Other AMPK family members including SIKs and Par-1/MARKs have also been implicated as either positive or negative regulators of the Hippo pathway^15,23–26^. The molecular mechanisms employed appear distinct, indicating an outstanding need for additional studies to fully appreciate the extent of overlap of biological function and molecular mechanism for all AMPK family members.

Kinases are considered imminently targetable for cancer therapeutics, but inhibiting the core kinase cassette components, MST/LATS, is not a viable option as this would act to promote tumor progression. Exploiting our identification of NUAK2 as a negative regulator of Hippo thus provides a new opportunity to develop kinase inhibitors that could counteract the oncogenic functions of YAP/TAZ.

## Methods

### Cell culture

The triple-negative breast cancer cell lines, MDA-MB231 and MDA-MB468, were cultured in RPMI and Dulbecco's modified Eagle's medium: nutrient mixture F-12 (DMEM:F-12) (1:1) supplemented with 5 and 10% fetal bovine serum (FBS), respectively. Human embryonic kidney 293T (HEK293T) cells were grown in DMEM supplemented with 10% FBS. The bladder cancer cell lines, T24 and HTB-2, were cultured in McCoy’s media and TCC and SW780 were cultured in RPMI supplemented with 10% FBS. The colon cancer cell line, SW480, was cultured in α-minimum essential medium supplemented with 10% FBS. Cell lines were obtained from ATCC and MDA-MB231, T24, and TCCSUP cell lines were authenticated by short tandem repeat analysis. Cell lines were monitored for mycoplasma contamination using the MycoAlert Mycoplasma Detection Kit (Lonza).

### High-throughput RNAi screen

The high-throughput RNAi (HT-RNAi) screen was carried out at the SMART Robotics Facility (http://nbcc.lunenfeld.ca). MDA-MB231 and MDA-MB468 cells, plated in 384-well dishes, were reverse transfected with 40 nM siRNAs derived from a siGENOME SMARTpool siRNA Human Kinome Library (G-003505, Dharmacon) using Lipofectamine RNAiMAX (Life Technologies). After 48 h, YAP/TAZ localization was visualized in cells co-stained with 4′, 6-diamidino-2-phenylindole dihydrochloride (DAPI) by automated immunofluorescence microscopy (IN Cell Analyzer 6000 Cell Imaging System). Subcellular localization was quantified by automated image analysis (Columbus software, PerkinElmer) and *Z*-scores for the nuclear-to-cytoplasmic ratio were calculated. Genes were ranked after taking the average score of two biological replicates for each cell line.

### Cloning, lentiviral infection, and cell-based assays

To introduce siRNAs (Dharmacon), cells were reverse transfected using Lipofectamine RNAiMAX (Life Technologies), while plasmids were delivered using Lipofectamine LTX (Life Technologies) according to the manufacturer's instructions. For serum switch and LPA (Avanti Polar Lipids) experiments, cells were starved overnight in media supplemented with 0.2% FBS and induced with fresh serum or LPA for the indicated times. For WZ4003 experiments, cells were treated with 10 μM WZ4003 (+) or dimethyl sulfoxide (DMSO) control (−) for 16 h or as indicated. For ON123300 experiments, cells were treated with the indicated concentration of ON123300 or DMSO control for 2 h or as indicated. The NUAK2 construct was generated using the Gateway system (Life Technologies) by transferring from a Gateway entry vector into C terminally tagged pCMV5-based destination vector. NUAK2 and LATS1 mutants and deletion constructs were generated by PCR-mediated site-directed mutagenesis. pCAGIP-Clover-YAP1 WT or 5SA was generated by assembly of multiple PCR fragments by overlapping PCR and NEBuilder assembly. Lentiviral NUAK2 short hairpin RNA (shRNA) plasmid was generated by cloning into pLV vector. For stable, inducible expression of NUAK2, the lentiviral pLD-puro-2A-rtTA-TcVA vector^53^ was used. The complementary DNA (cDNA) fragment encoding DN c-JUN was PCR cloned from pMIEG3-JUN DN^54^ obtained from Addgene (plasmid #40350) into a Gateway entry vector and then transferred into pLEX_307 provided by D. Root to Addgene (plasmid #41392). For lentiviral production, HEK293T cells were co-transfected with the DN c-JUN and packaging plasmids using Lipofectamine 2000 (Life Technologies). Viral supernatants were collected 48 h post transfection and MDA-MB231 cells were infected using polybrene. The siRNA oligos are listed in Supplementary Table 2.

### Generation of stable cell lines

*NUAK2* KO MDA-MB231 cells were generated using CRISPR/Cas9 by transfecting cells with plasmids pSpCas9(BB)-2A-Puro (PX459)^55^ obtained from Addgene (plasmid #48139) encoding two guide RNAs targeting exon 1 of *NUAK2* (TGGAGTCGCTGGTTTTCGCG, GGGTCTCCAGGAACTCGTAG). Positive cells were selected using puromycin for 2 days starting 33 h post transfection. Cell clones lacking NUAK2 expression were identified by immunoblotting with anti-NUAK2 antibodies and by assessing NUAK2 mRNA expression levels by qPCR using primers targeting exon 1. Stable cell clones overexpressing WT or 5SA variants of Clover-YAP were generated in MDA-MB231 cells by puromycin selection. For lentiviral constructs, viral supernatants were generated by co-transfecting HEK293T cells with pLD-NUAK2 or NUAK2 shRNA constructs along with packaging plasmids using Lipofectamine 2000 (Life Technologies). MDA-MB231 or TCCSUP or T24 cells were infected using polybrene and stable cell pools or clones were selected with puromycin.

### Immunoblotting, immunoprecipitation, and immunofluorescence microscopy

Cell lysates were analyzed directly or subjected to immunoprecipitation prior to immunoblotting using standard protocols^31,56^. Samples were separated on regular sodium dodecyl sulfate-polyacrylamide gel electrophoresis (SDS-PAGE) or on Phos-Tag gels, using reagents from Waco Chemicals. Full size immunoblots are shown in Supplementary Figs. 1,1 and 12. To quantitate the amount of phosphorylated to non-phosphorylated YAP or TAZ on Phos-Tag gels, the ratio of the slowest migrating bands to the fastest (non-phosphorylated) was determined using Image J. For immunofluorescence microscopy, cells were fixed with 4% paraformaldehyde, permeabilized with 0.5% Triton X-100/phosphate-buffered saline (PBS) and blocked with 2% bovine serum albumin/PBS. A minimum of 30 cells per experimental condition were quantitated and data are plotted as the percentage of cells with equivalent nuclear and cytoplasmic (N = C), predominantly nuclear (N > C), or predominantly cytoplasmic (C > N) YAP/TAZ localization.

### Antibodies for immunoprecipitation, immunoblotting, ChIP, and immunofluorescence

For immunoprecipitation, the following antibodies were used: mouse anti-Flag M2 (F1804, Sigma-Aldrich, 1:1000), IgG (2729, Cell Signaling Technology), and LATS1 (C66B5, Cell Signaling Technology, 1:100). For immunoblotting, primary antibodies used were: YAP (sc-101199, Santa Cruz, 1:1000), p-YAPS127 (D9W2I, Cell Signaling Technology, 1:1000), TAZ (560235, BD Biosciences, 1:1000), LATS1 (C66B5, Cell Signaling Technology, 1:3000), NUAK2 (NBP1-81880, Novus, 1:200), rat anti-HA (1867423, Roche, 1:1000), and anti-Flag M2 (F1804, Sigma-Aldrich, 1:3000). For secondary antibodies, horse radish peroxidase-linked anti-mouse or anti-rabbit antibodies (Santa Cruz, 1:10,000) were used. For ChIP the following antibodies were used: YAP/TAZ (D24E4, Cell Signaling Technology, 1:50), TEAD (ab58310, Abcam, 2.5 μg/ChIP), c-JUN (610326, BD Biosciences, 2.5 μg/ChIP), and IgG (I5006, Sigma-Aldrich, 2.5 μg/ChIP). For immunofluorescence, the primary antibodies used were: YAP1 (sc-101199, Santa Cruz, 1:300), E-cadherin (610181, BD Biosciences, 1:500), and rabbit anti-Flag (F7425, Sigma-Aldrich, 1:500), and secondary antibodies used were: goat anti-rabbit Alexa Fluor 488 (A11305, Life Technologies, 1:1000), goat anti-mouse Alexa Fluor 546 (A11029, Invitrogen, 1:1000), and donkey anti-mouse CF555 (20037, Biotium, 1:1000). Samples were counterstained with DAPI (D9542, Sigma-Aldrich) and Alexa Fluor 488-phalloidin (A12379, Thermo Fisher Scientific, 1:500 dilution) was used for actin cytoskeleton staining.

### Live cell imaging and time-lapse imaging

Live cell imaging of Clover-YAP-expressing MDA-MB231 cells was carried out using a custom WAVE-FX-X1 spinning disc confocal system (Quorum Technologies) with a modified Yokogawa CSU-X1 scanhead on an AxioObserver Z1 inverted microscope (Carl Zeiss) with a ×40 NA 1.2 Plan Apochromat (Carl Zeiss) objective. Cells were plated in a 35 mm glass-bottom dish (Mat-Tek, P35G-1.5-14-C) and were maintained in a stage-top incubator at 37 °C and 5% CO_2_ during imaging. Cells were cultured in phenol-red free RPMI medium (Thermo Fisher Scientific, 11835030) with 5% FBS and 200 nM SiR-DNA (Spirochrom, SC007) was added 1 h prior to imaging to visualize the nuclei. Localization of Clover-YAP was monitored every 10 min for 2 h. Volocity software was used for image acquisition and processing.

### RNA extraction and Real-Time PCR

Total RNA was extracted from cultured cells using PureLink RNA Mini Kit (Life Technologies) and reverse transcribed into cDNA using oligo(dT) primers and M-MLV Reverse Transcriptase (Invitrogen). Real-time PCR was performed on the ABI Prism 7900 HT system (Applied Biosystems) using SYBR Green master mix (Applied Biosystems). Relative gene expression was quantified by ΔΔC_t_ method and normalized to HPRT1. The primer sequences are listed in Supplementary Table 2.

### Chromatin immunoprecipitation-qPCR

MDA-MB231 cells that were starved overnight (0.2% FBS) and then stimulated with complete media for 1 h were subjected to ChIP-qPCR. Cells were crosslinked with 1% formaldehyde in PBS for 10 min at room temperature and chromatin from lysed nuclei was sheared, incubated with YAP, TEAD, or c-JUN antibodies at 4 °C for 4 h and then collected using proteinG Dynabeads. DNA was purified and analyzed by qPCR. Binding of TEAD, YAP, c-JUN, or IgG control to the *NUAK2* or *ANKRD1* enhancer sequences was plotted as a fraction of DNA input. Primer sequences are listed in Supplementary Table 2.

### Luciferase reporter assays

The TEAD-luciferase reporter WT construct is comprised of 10 tandem TEAD binding sites that drive the expression of the luciferase gene, while the mutant variant (MUT) harbors mutations in all TEAD binding sites^35,57^. Cells were transfected with TEAD-luciferase and β-galactosidase at 24 h post-siRNA transfection. After 24 h, cells were lysed in lysis buffer (25 mM Tris, 2 mM dithiothreitol (DTT), 2 mM 1,2-diaminocyclohexane *N*,*N*,*N*′,*N*′-tetraacetic acid (DCTA), 10% glycerol, 1% Triton X-100) and luciferase and β-galactosidase activity measured using MicroLumatPlus-LB96V (EG&G Berthold) and Emax (Molecular Devices) instruments, respectively. Luciferase activities were normalized to β-galactosidase activity.

### LUMIER screen

For the LUMIER protein–protein interaction screen^58,59,60^, the interaction of Luciferase-tagged versions of TAZ and kinase dead LATS1/2 and MST1/2 were screened against a library of Flag-tagged proteins that included NUAK2, and association with the immunopreciptated Flag-NUAK2 was detected by luciferase assay. Screening results are plotted as fold over median^31,32,59^ (median luciferase interaction ratio).

### In vitro kinase assays and mass spectrometry

HEK293T cells were transfected with Flag-LATS1 alone or together with MST1. Protein Sepharose beads containing immunoprecipitated LATS1 were washed three times with wash buffer and twice with kinase assay buffer (50 mM HEPES, pH 7.5, 100 mM NaCl, 5 mM, MgCl_2_, 2 mM DT). The immunoprecipitated LATS1 was pre-incubated with purified MOB1A T353 (MOB1A*) activated by in vitro phosphorylation by MST^17,34^ unless otherwise indicated, with or without 0.5 μg baculovirally produced NUAK2 (SignalChem) for 30 min at 30 °C and then subjected to a kinase assay for 30 min at 30 °C in the presence of 150 μM cold ATP, 10 μCi [γ-^32^P]ATP and 20 μM of bacterially produced GST-YAP (50-171) peptide. Reactions were terminated with SDS sample buffer and separated by SDS-PAGE. Phosphorylation was visualized by phosphorimaging and protein levels were determined by Coomassie blue staining. To examine NUAK2-mediated phosphorylation of LATS, the immunoprecipitated LATS1 was incubated with 0.05 μg baculovirally produced NUAK2 (SignalChem) and was subjected to a kinase assay for 2.5 h at 30 °C in the presence of 150 μM cold ATP and 10 μCi [γ-^32^P]ATP. To identify NUAK2 targeted sites, phosphopeptides derived from in vitro phosphorylated LATS incubated with NUAK2 in the presence or absence of ATP from one experiment was determined by mass spectroscopy. To identify cell-based NUAK2 target sites, HEK293T cells were co-transfected with Flag-LATS1, MOB1A-HA alone or together with NUAK2-HA WT, or kinase dead (K81R). Phosphopeptides derived from immunoprecipitated LATS1 were determined by mass spectroscopy. To examine the effect of NUAK inhibitors, HEK293T cells transfected with Flag-LATS1 were pre-treated with DMSO (control), WZ4003, or ON123300 (10 μM) for 4 h prior to immunoprecipitation. Samples were analyzed by liquid chromatography with tandem mass spectrometry using parallel reaction monitoring to measure LATS1 peptides. LATS1 phosphopeptide values were normalized across samples by using signals from LATS1 peptides not subject to phosphorylation^61^. The tryptic peptide 609-QITTpSPITVR-618 displayed an anomalously increased reverse phase chromatography elution time >7 min compared with its non-phosphorylated counterpart. Adjacent lysines at 607–608 were subject to consistent tryptic cleavage limitation^62^. Therefore, peptide 608-KQITTpSPITVR-618, which eluted approximately 3 min sooner than the cognate non-phosphorylated peptide, was used for estimations of S613 phosphorylation.

### Orthotopic mouse model and tissue immunostaining

All mouse experiments were conducted in accordance with protocols approved by the animal facility at Toronto Centre for Phenogenomics as approved by the Canadian Council on Animal Care. MDA-MB231 cells (1.5 × 10^6^) comprised a pool of three clones of *NUAK2* KO (*n* = 8 mice per group) or empty vector controls (*n* = 7 mice per group) were inoculated into the right, fourth mammary fat pad of 10-week-old female C.B-17 severe combined immunodeficiency mice and tumor growth was measured weekly^63,64^. For immunofluorescent staining, MDA-MB231 tumors were harvested from mice sacrificed at 3 weeks and fixed in 10% buffered formalin phosphate (SF100-4, Fisher Scientific) prior to paraffin embedding. Tissue sectioning was performed at 4 mm thickness. The primary antibodies used for immunostaining were: rabbit anti-Yap (14074, Cell Signaling, 1:100) and mouse anti-human vimentin (M0725, Dako, 1:100) to identify MDA-MB231 cells.

### Cell growth assays

Cell growth was determined using either the sulforhodamine B (SRB) assay or by DAPI staining. Cells were plated overnight in 96-well plates and then cultured in RPMI supplemented with 1% FBS or 10% FBS with fresh media addition each day. For the SRB assay, cells were fixed with 10% (weight/volume) trichloroacetic acid and stained with 0.4% (w/v) SRB^65^ at 24, 48, and 72 h post-siRNA transfection or WZ4003 treatment. The amount of SRB present in each well (*n* = 6 per condition) was determined by optical density reading at 490 nm. For DAPI staining, cells were fixed with 4% paraformaldehyde, permeabilized with 0.5% Triton X-100/PBS, stained with DAPI to visualize nuclei by using automated immunofluorescence microscopy (IN Cell Analyzer 6000 Cell Imaging System), and quantified by automated image analysis (Columbus software, PerkinElmer).

### Gene expression and statistical analysis

NUAK1 and NUAK2 expression in bladder cancer patients was analyzed using previously published datasets^39–41^. For the Hedegaard et al. NMIBC dataset^40^, data were filtered to remove samples with low-quality RNA prior to analysis. To establish the YAP Signature Genes score (YAP SG), the RNAseq transcriptional profiles (GEO Accession: GSE59483) of 49 bladder cancer patient samples from Liu et al.^39^ were used. For this, *Z*-scores for each individual gene in the previously established cancer-associated YAP signature^13^, also listed in Supplementary Table 1, were summed^66^. Correlation of NUAK2 expression to the YAP SG was determined using Pearson’s correlation coefficient and significance was determined by comparison to 1300 permutations of randomly selected 57 gene sets. All other statistics were calculated using Student’s unpaired two-sided *t* test. Sample size was not predetermined and the investigators were not blinded to the group allocation during experiments and outcome assessment. To generate heat maps, average linkage hierarchical clustering with an uncentered correlation was performed using Cluster 3.0 and visualized using Java Tree View.

## Electronic supplementary material

ANKRD1 has been made roman)"?>Supplementary Information

Description of Additional Supplementary Files

Supplementary Movie 1

Supplementary Movie 2

## Data Availability

All data generated or analyzed during this study are included in this published article and its Supplementary Information files.
