# Supplementary material for: A feed forward loop enforces YAP/TAZ signaling during tumorigenesis
Source: Nat Commun. 2018 Aug 29;9:3510. doi: 10.1038/s41467-018-05939-2 (PMC6115388; doi:10.1038/s41467-018-05939-2)
Supplement: Supplementary file 1 — ANKRD1 has been made roman)"?>Supplementary Information [file 41467_2018_5939_MOESM1_ESM.pdf]

SUPPLEMENTARY INFORMATION

for

A feed forward loop enforces YAP/TAZ signaling during tumorigenesis.

Mandeep K. Gill, et al

SUPPLEMENTARY TABLE OF CONTENTS

Supplementary Figures 1-12 and Figure Legends.

Supplementary Table 1 and 2.

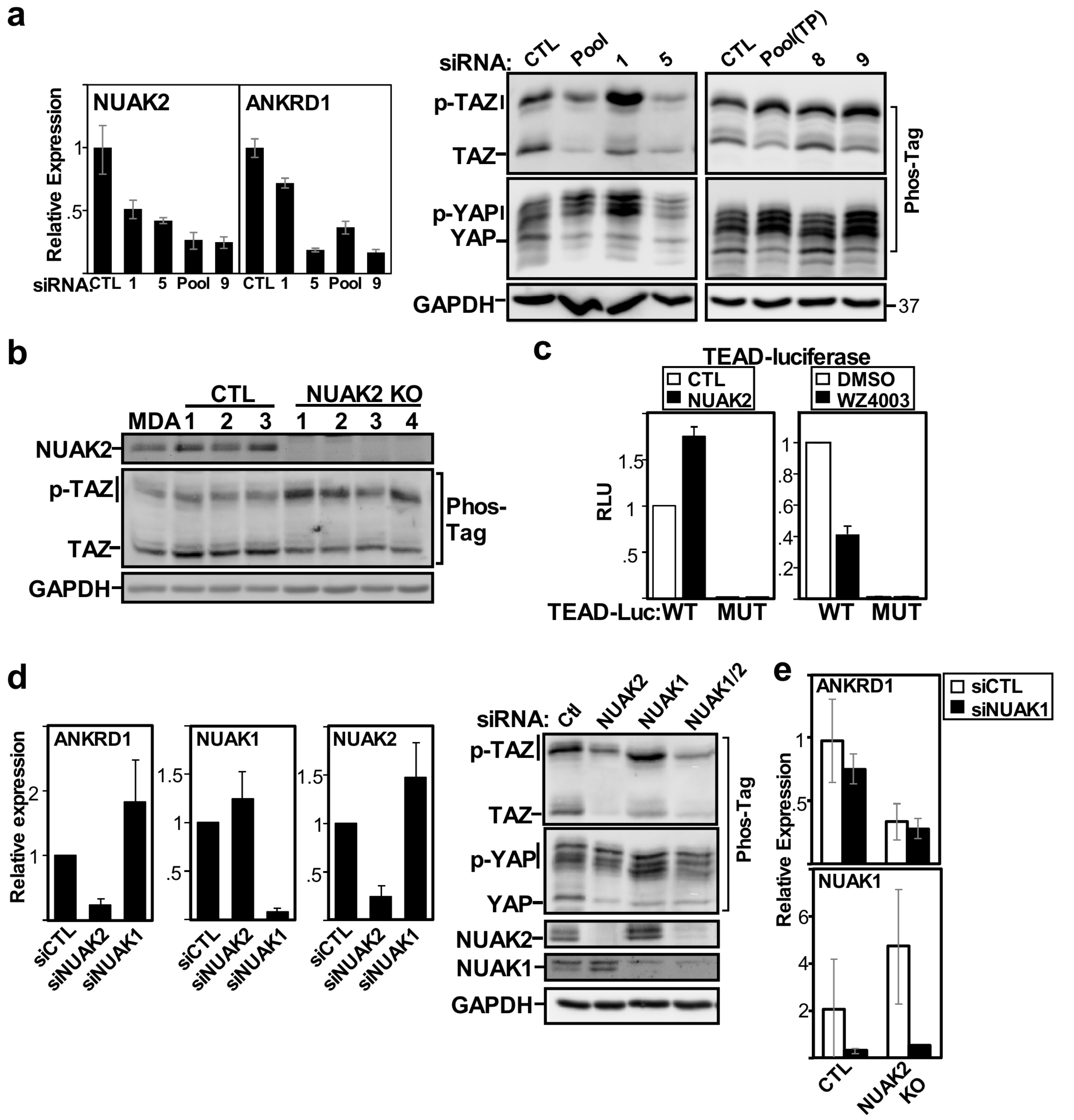

Supplementary Figure 1. Examination of the function of NUAK1 and NUAK2 on YAP/TAZ phosphorylation, localization and target gene expression.

(a) Loss of NUAK2 expression using pooled or single siRNAs represses YAP/TAZ target gene expression (left panel) and increases phosphorylation of YAP/TAZ (right panel) in MDA-MB231 cells. Data (left) is plotted as the mean +/- the range of a representative experiment. Pool: siGENOME pool; Pool(TP): ON-TARGET plus siRNA pool. **(b)** *NUAK2* KO in MDA-MB231 cells increases phosphorylation of TAZ. **(c)** Overexpression of NUAK2 increases (left) and treatment with **WZ4003** (10 µM) for 16 h suppresses (right) TEAD reporter activity in MDA-MB231 cells. Data is plotted as the mean +/- SD (n=3). **(d, e)** Analysis of ANKRD1 expression upon abrogating NUAK1 or NUAK2 expression using siRNAs in MDA-MB231 cells (d, left) plotted as the mean +/- SD, n=5 (MDA-MB231) or in MDA-MB231 *NUAK2* KO cells (e) where a representative experiment is plotted as the mean +/- SD, of three individual clones for each of CTL and *NUAK2 KO* lines. (d) Loss of NUAK2 but not NUAK1 in MDA-MB231 cells increases YAP/TAZ phosphorylation as assessed using PhosTag gels (right panel).

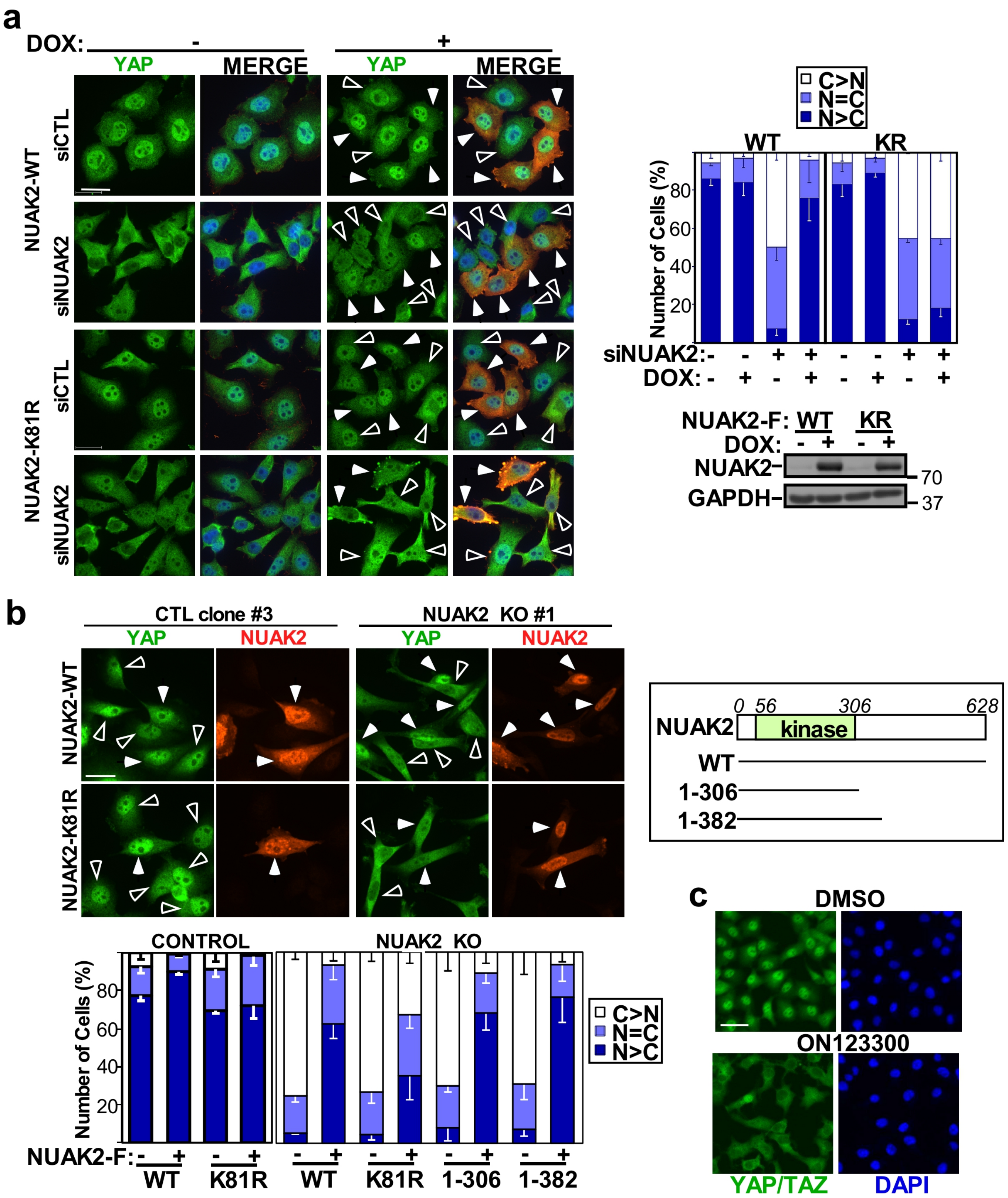

Supplementary Figure 2. NUAK2 kinase activity is required to rescue the siNUAK2 or *NUAK2* KO phenotype. (a) MDA-MB231 cell pools stably-expressing Dox-inducible wild type (WT) or kinase-deficient (K81R) NUAK2-Flag were transfected with siNUAK2 and YAP/TAZ localization in the presence or absence of Dox (1 μg/l) was examined by immunofluorescence microscopy. **(b)** MDA-MB231 control (CTL) or *NUAK2* KO clones were transfected with NUAK2-Flag wild type (WT), mutant (K81R: kinase-dead) or deletion constructs as shown in the schematic. (a,b) Localization of endogenous YAP (green) in NUAK2-Flag expressing (red; white arrows) or non-expressing (arrowheads) cells with nuclei co-stained with DAPI (blue) is shown. Quantitation is plotted as the mean +/- SD, n=3 (a), or from three individual clones for each NUAK2-Flag construct tested in two independent experiments (b). Scale bar, 25 μm. N, nuclear; C, cytoplasmic. **(c)** Treatment of MDA-MB231 cells with **ON123300** (10 μM) for 2 h promotes cytoplasmic localization of YAP/TAZ.

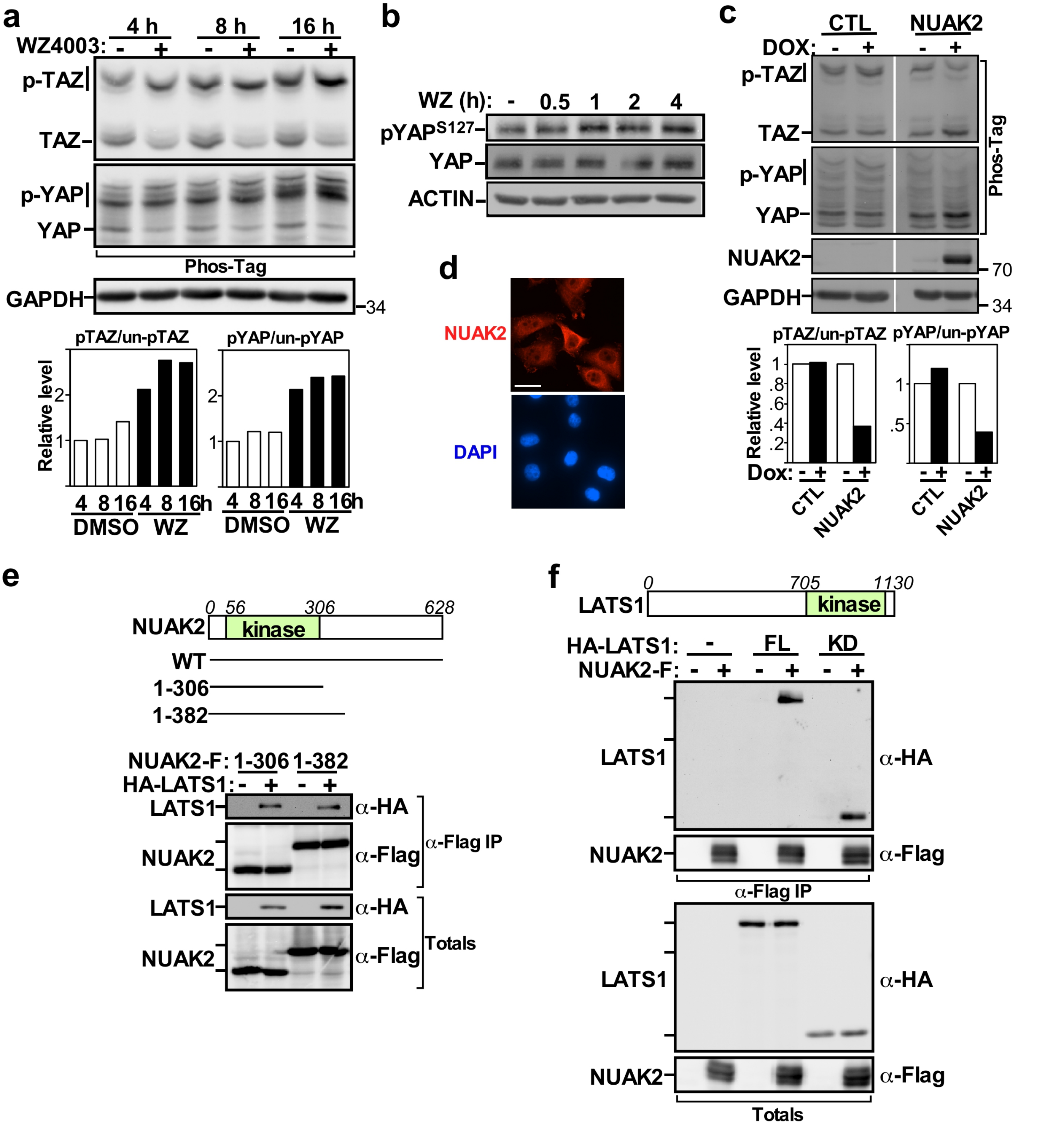

Supplementary Figure 3. NUAK2 interacts with LATS and regulates YAP/TAZ phosphorylation.
(a, b) Blocking NUAK2 with **WZ4003** (10 μM) increases YAP/TAZ phosphorylation in MDA-MB231 cells as assessed on PhosTag gels (a) and quantitated from blots (bottom) or using phospho-YAPS127 antibodies (b). **(c)** Dox-induced overexpression of NUAK2-Flag in MDA-MB231 stables decreases phosphorylation of YAP/TAZ (top). Relative phosphorylation levels from blots is quantitated (bottom). Note the low level of NUAK2 expression in the absence of induction. **(d)** NUAK2, visualized with anti-Flag antibodies is localized to the cytoplasm in NUAK2-Flag overexpressing MDA-MB231 inducible stables. **(e, f)** The kinase domains of LATS1 and NUAK2 are sufficient for interaction. HEK293T cells were co-transfected with NUAK2-Flag or HA-LATS1 alone or together with mutant constructs of HA-LATS1 (e) or NUAK2-Flag (f). FL: full length, KD: kinase domain. Cell lysates were subjected to anti−Flag immunoprecipitation, and the presence of LATS1 was determined by anti−HA immunoblotting. Schematics depicting the kinase domains of LATS1 and NUAK2 are shown.

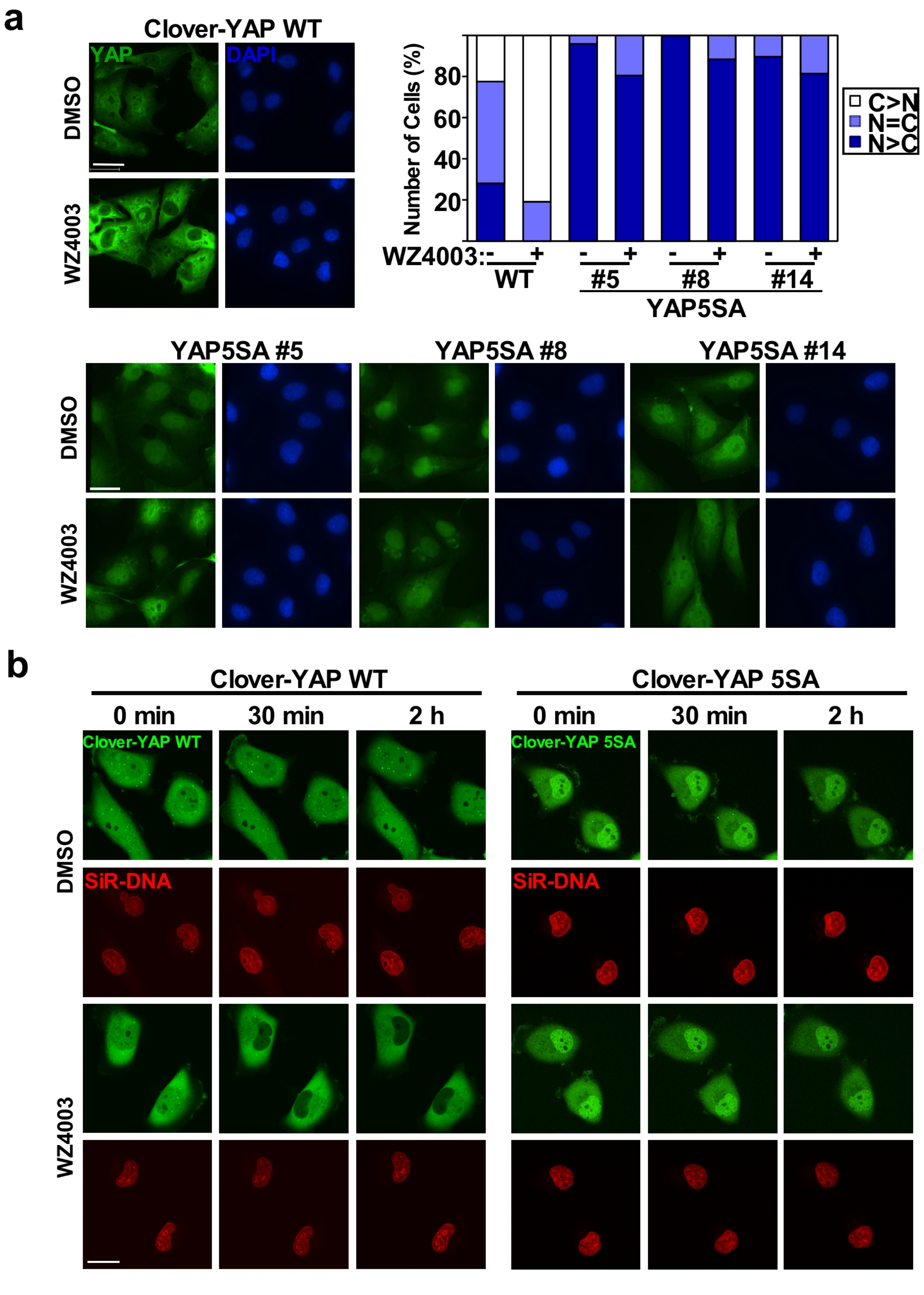

Supplementary Figure 4. YAP mutated in the LATS-targeted phosphorylation sites are resistant to the effects of the NUAK inhibitor, WZ4003.
(a) Inhibition of NUAK2 activity by **WZ4003** has no effect on the localization of a phosphorylation site mutant variant (5SA) of YAP. MDA-MB231 cells stably expressing wild type (WT) or phosphorylation site mutant (5SA) versions of Clover-tagged YAP were treated **WZ4003** (10 μM) or DMSO control for 16 h. Localization of WT and 5SA YAP (green) cells with nuclei co-stained with DAPI (blue) in fixed cells was visualized by immunofluorescence confocal microscopy. Quantitation of a representative experiment is plotted (a, top right).
(b) Time-lapse imaging of Clover-YAP in MDA-MB231 cells. Localization of Clover-YAP wild type or 5SA (green) was monitored after addition of **WZ4003** (10 μM) in cells with nuclei (red) visualized with SiR-DNA. Optical section images at the indicated time show cytoplasmic accumulation of YAP upon addition of **WZ4003** in WT but not 5SA-expressing cells. Scale bar, 25 μm.

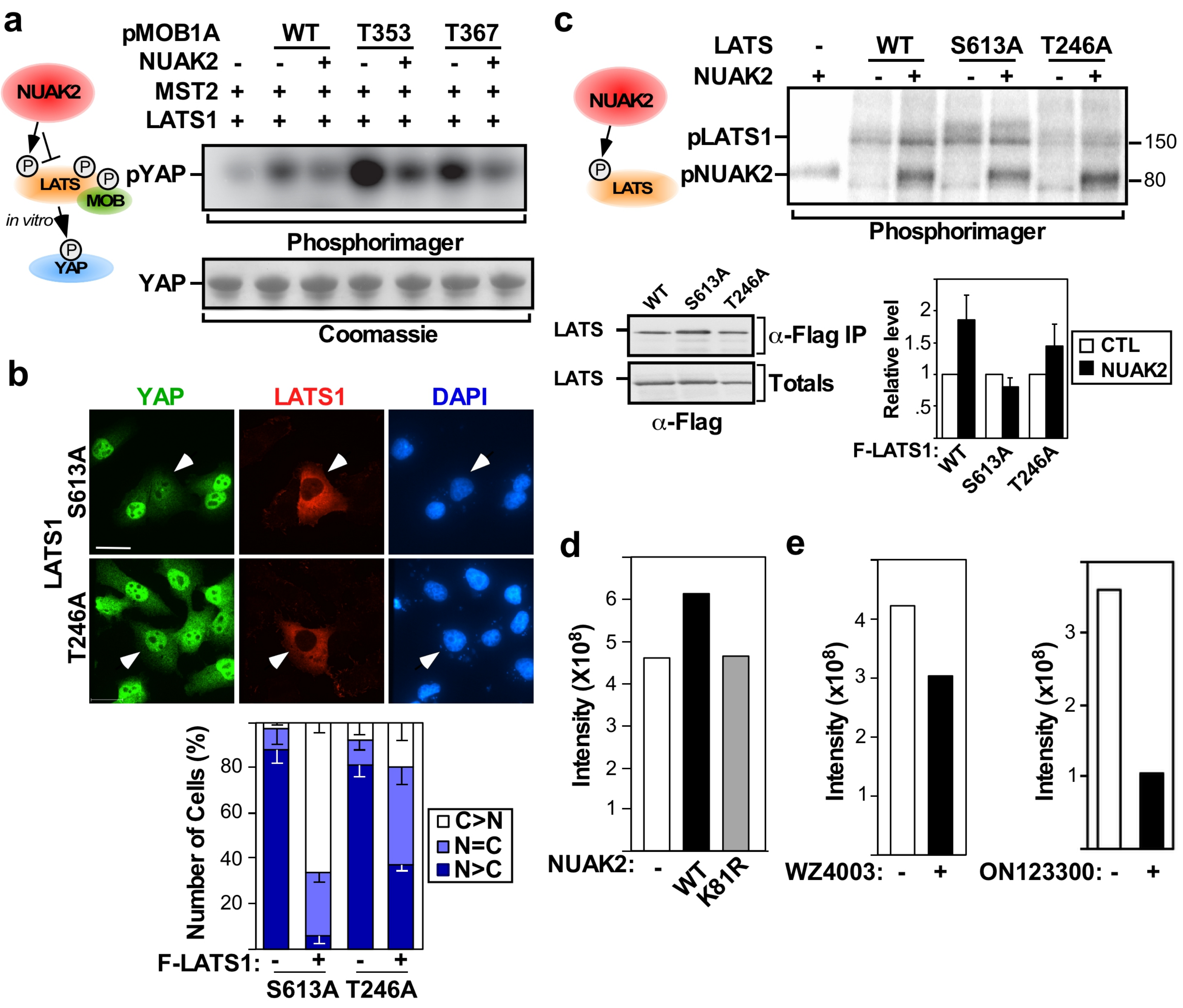

Supplementary Figure 5. NUAK2 phosphorylates and inhibits LATS activity.

(a) Analysis of LATS-mediated phosphorylation of YAP in an in vitro kinase assay. Addition of variants of MOB1A, T12 to T353 (T353) or T12 to T367 (T367) previously phosphorylated by MST in vitro enhances LATS activity towards YAP, all of which are inhibited by NUAK2. **(b)** Analysis of the effects of LATS S613A and T246A single mutants on cytoplasmic localization of YAP. Localization of endogenous YAP (green) in transfected (white arrows) or non-transfected cells, with NUAK2-Flag (red, marked with white arrows) and nuclei co-stained with DAPI (blue) are shown. Quantitated results are plotted as the mean +/- SD (n=3). Scale bars, 25 µm. N, nuclear; C, cytoplasmic. **(c)** Analysis of NUAK2-mediated phosphorylation of WT and single S613A or T246A mutants of LATS in an in vitro kinase assay. Levels of immunoprecipitated LATS WT (wild type), and single mutants T246A or S613A are comparable. Relative phosphorylation levels from blots is quantitated and plotted as the mean +/- SD (n=2) (right). **(d, e)** Overexpression or inhibition of NUAK2 activity modulates pSer613 levels in mammalian cells. LATS was isolated by immunoprecipitation and samples analyzed by LC-MS/MS using parallel reaction monitoring (PRM) to measure LATS1 peptides. LATS1 phospho-peptide values were normalized across samples using signals from LATS1 peptides not subject to phosphorylation. (d) HEK293T cells were transfected with Flag-LATS, HA-MOB1A in the presence or absence of WT or Kinase-deficient (KR) NUAK2. A representative result from two independent experiments is shown. **(e)** Flag-LATS transfected HEK293T cells were treated with for 4 h with **WZ4003** (10 µM) or **ON123300** (10 µM) prior to lysis.

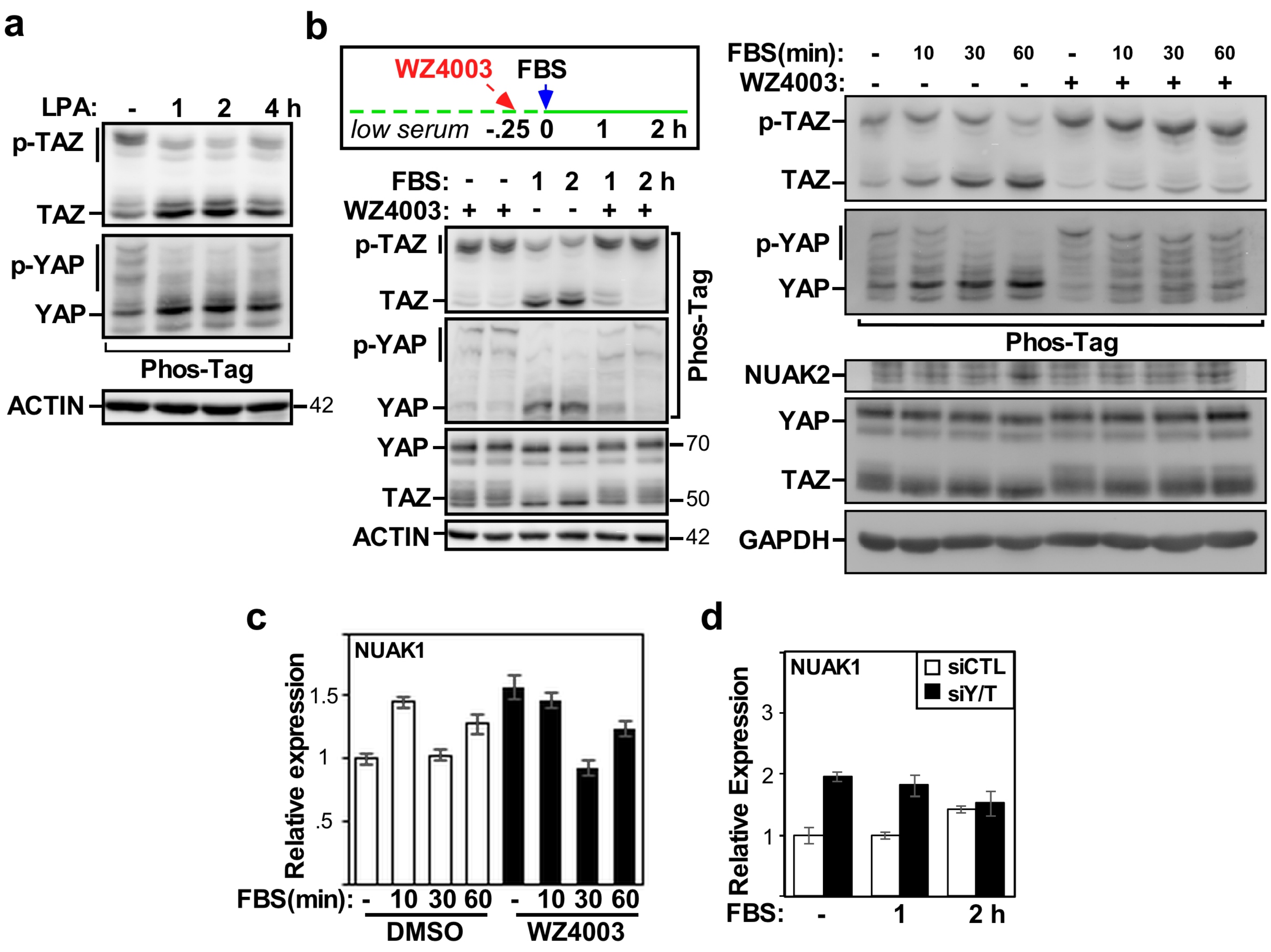

Supplementary Figure 6. YAP/TAZ regulates NUAK2, but not NUAK1 in a positive feed forward loop in MDA-MB231 cells.

(a) LPA induces YAP/TAZ dephosphorylation. **(b, c)** Pretreatment with **WZ4003** (10 μM) for the indicate times inhibits FBS induced YAP/TAZ dephosphorylation and NUAK2 protein levels as monitored on PhosTag or regular SDS-PAGE gels, respectively. **(d)** Treatment of cells with FBS, **WZ4003** or siYAP/TAZ does not alter NUAK1 mRNA expression as determined by qPCR. Data is plotted as the mean +/- the range of a representative experiment.

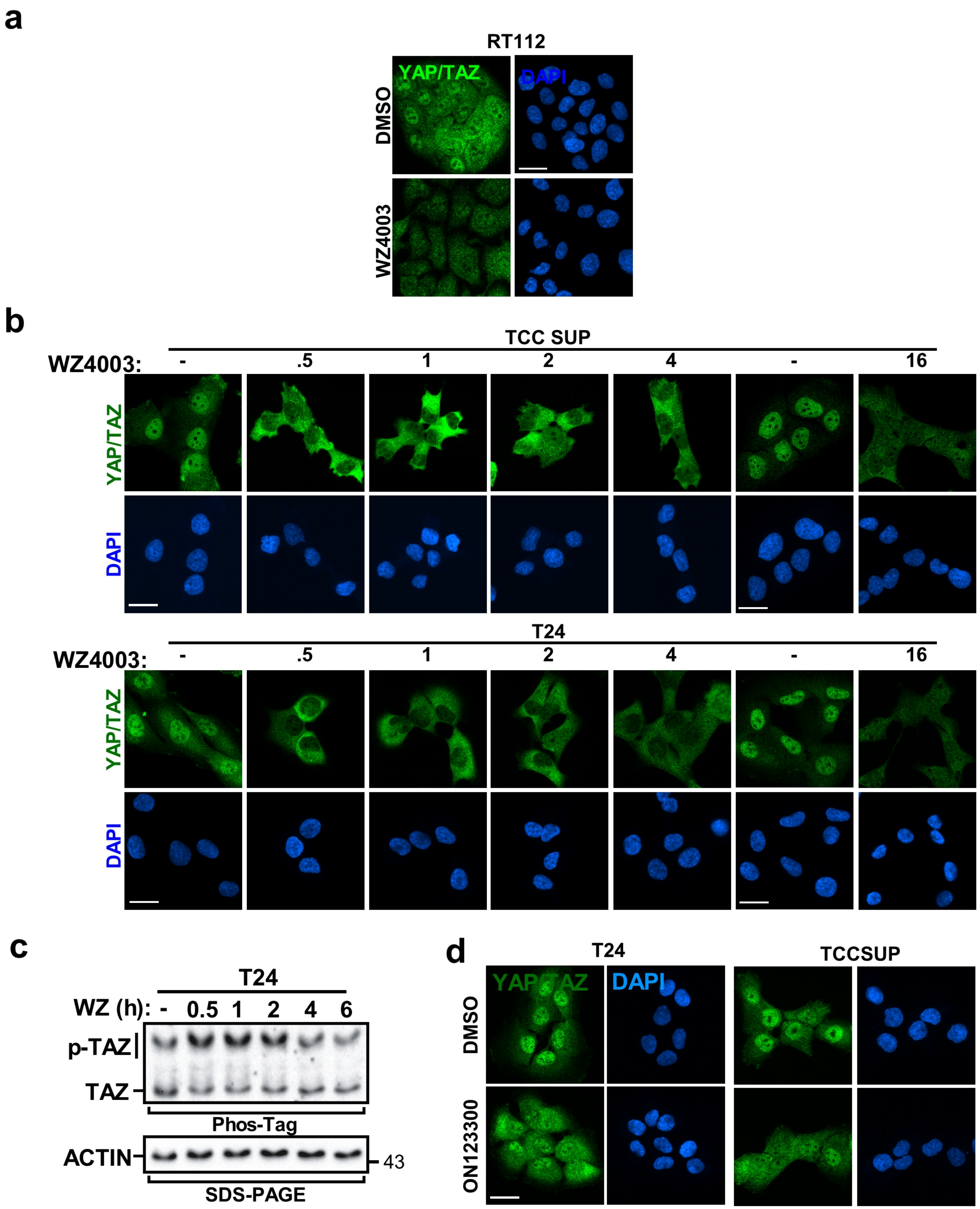

Supplementary Figure 7. Blocking NUAK2 induces cytoplasmic accumulation of YAP/TAZ in bladder cancer cells.
(a-c) Treatment of the indicated bladder cancer cell lines with **WZ4003** (10 µM) for 16 h (a) or from 30 min to 4 h (b,c) promotes cytoplasmic localization (a,b) and induces phosphorylation (c) of YAP/TAZ. Note that total YAP/TAZ levels are reduced upon prolonged (16 h) incubation (b). **(d)** Treatment of T24 and TCCSUP cells for 4 h with **ON123300** at 100 nM or 500 nM respectively, promotes cytoplasmic localization of YAP/TAZ. Scale bars, 25 µm.

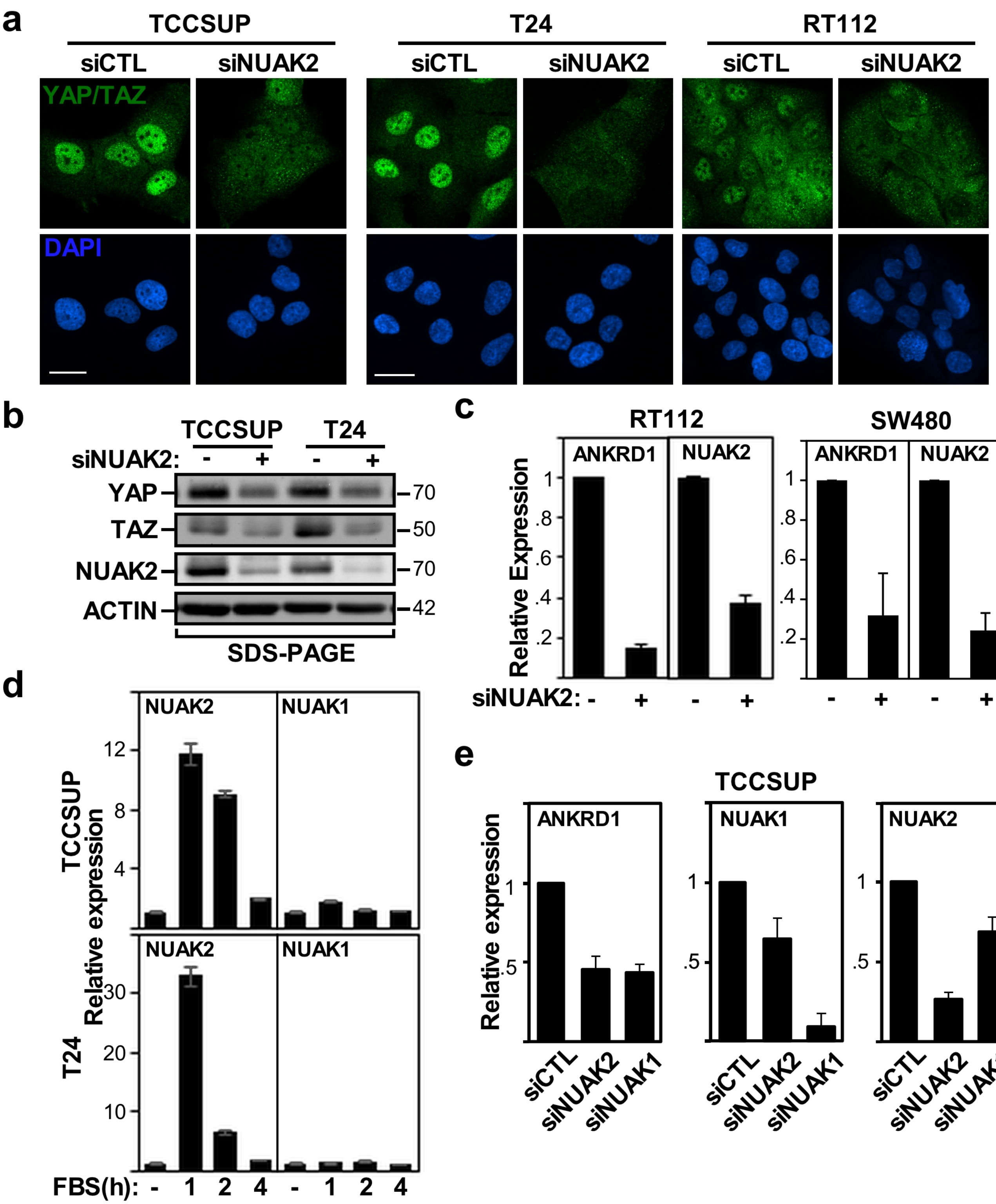

Supplementary Figure 8. Blocking NUAK2 expression inhibits YAP/TAZ function.
(a-b) Loss of NUAK2 expression in bladder cancer (TCCSUP, T24 and RT112) cells decreases YAP/TAZ protein levels as visualized by immunofluorescence microscopy (a) and immunoblotting (b). **(c)** Loss of NUAK2 expression represses YAP/TAZ target gene expression in RT112 and SW480 cells. Data is plotted as the mean +/- SD (n=3). **(d)** NUAK1 expression is not induced by FBS in T24 or TCCSUP cells. Data is plotted as the mean +/- range of a representative experiment. **(e)** Analysis of ANKRD1 expression upon abrogating NUAK1 or NUAK2 expression using siRNAs in TCCSUP cells is plotted as the mean +/- SD, n=3. Note that in TCCSUP cells, siNUAK1 also causes a reduction in the levels NUAK2.

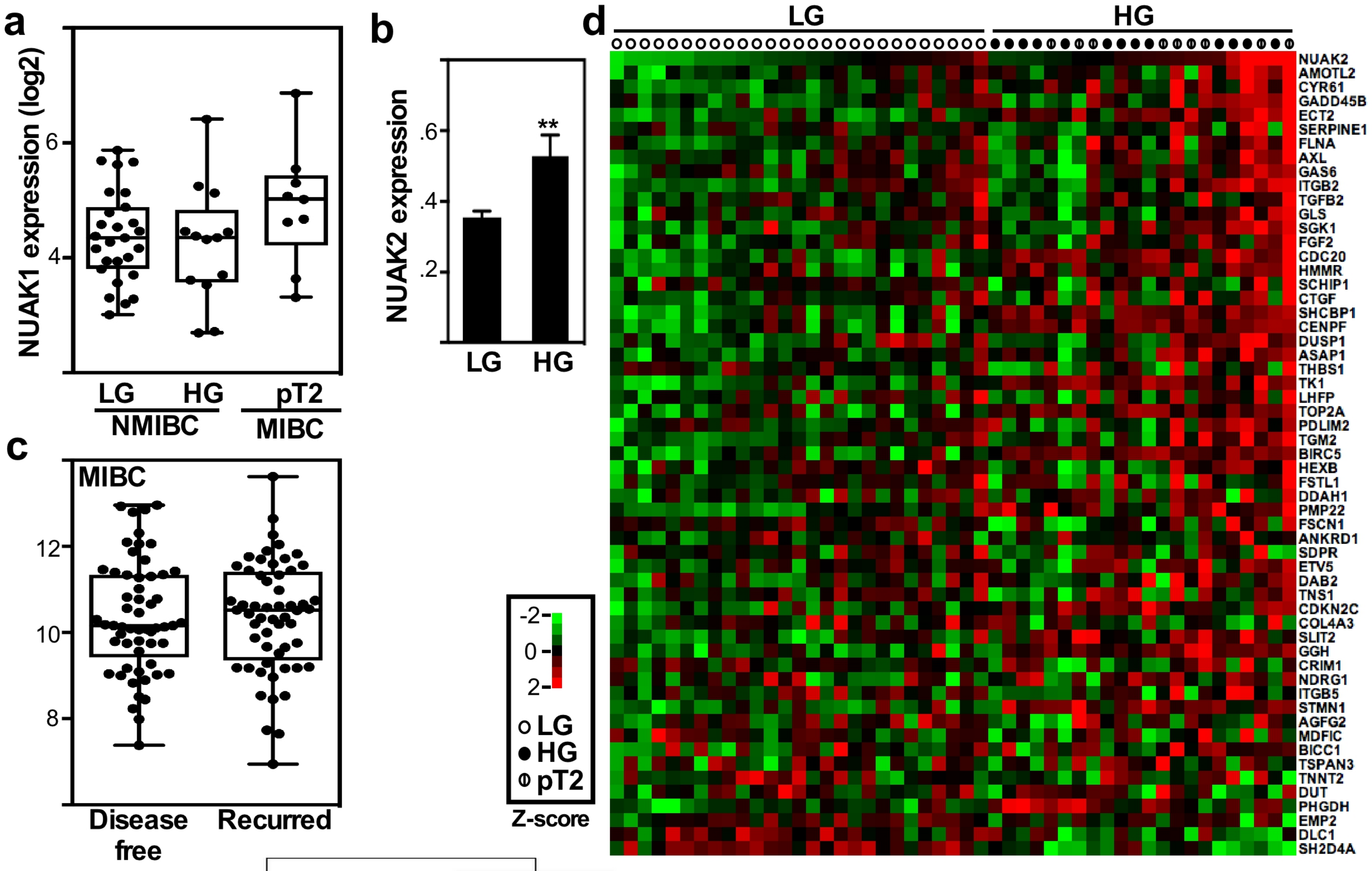

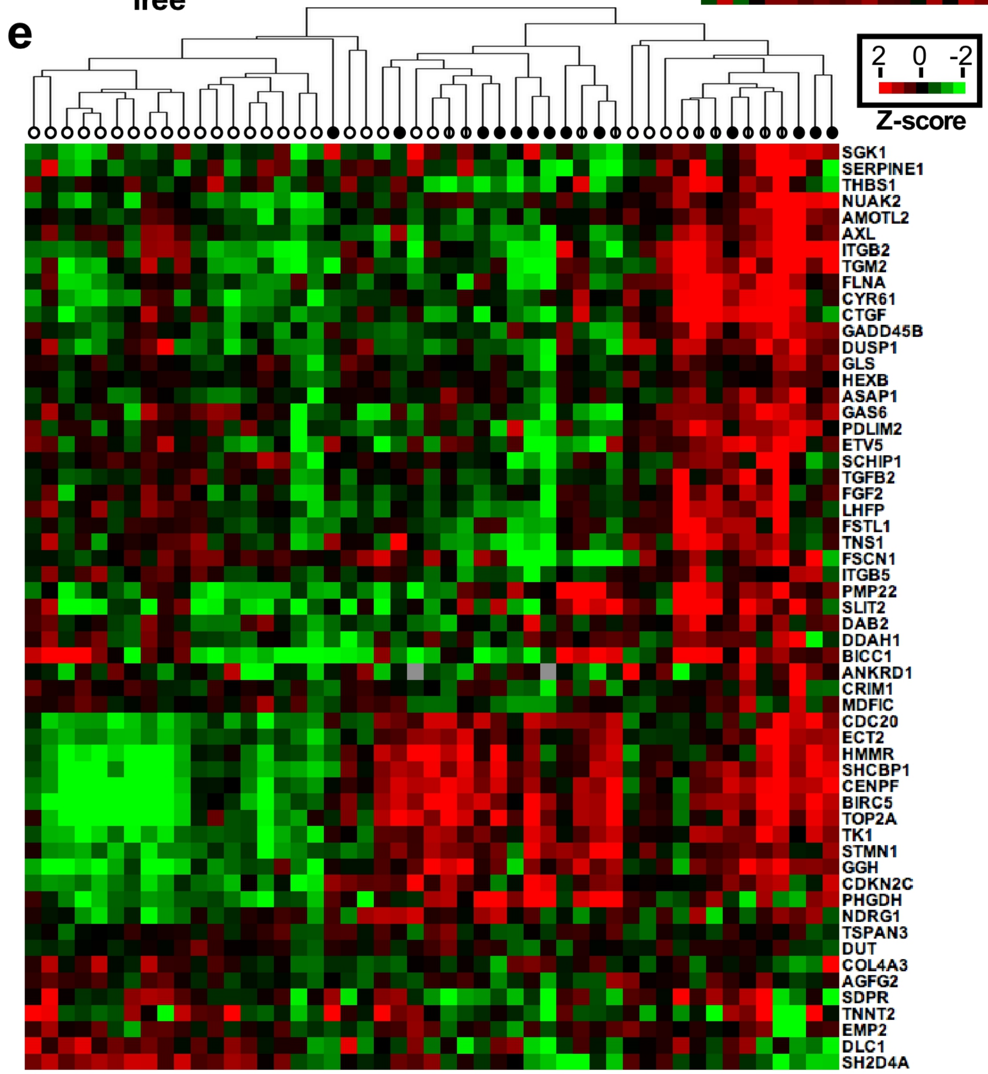

Supplementary Figure 9. NUAK2 expression correlates with a YAP gene signature in bladder cancer patient samples.

(a) Expression of NUAK1 is not significantly altered in HG NMIBC (HG NMIBC; n = 13, p=0.9979) or MIBC (pT2 MIBC; n = 9, p=0.0778) bladder cancers as compared to LG NMIBC (n = 27) using an unpaired t-test. **(b)** Expression of NUAK2 is elevated in HG versus LG disease in a NMIBC patient cohort with a mean of 0.5285 (n=192) versus 0.3549 (n=284), respectively (**p value = 0.0014). **(c)** Expression of NUAK1 is not correlated with disease recurrence in a cohort of MIBC patient samples with n= 57 disease free and n= 56, recurred samples (p=0.9135). Box plots are plotted as in Fig. 7. **(d, e)** Heat maps depicting expression levels of NUAK2 and individual YAP signature genes (d) or hierarchical clustering of NUAK2 and individual YAP signature genes (e) in LG (open circle) and HG NMIBC (filled circles) or MIBC (pT2) (slashed circles) bladder cancer samples.

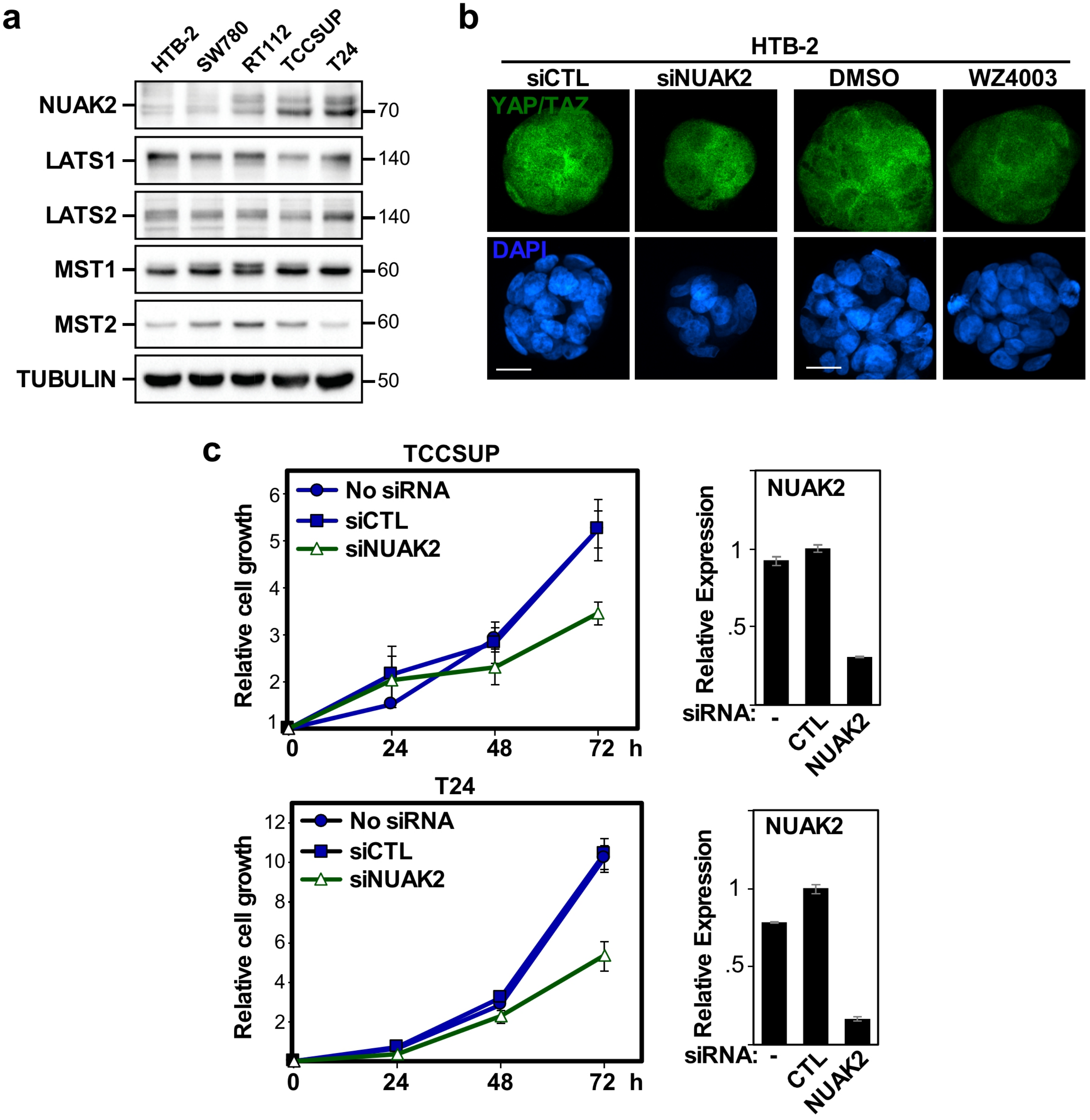

Supplementary Figure 10. Blocking NUAK2 attenuates growth in bladder cancer cells.
(a) Total protein levels in the indicated bladder cancer cell lines were visualized by immunoblotting. **(b)** Loss of NUAK2 by siRNA or by treatment with **WZ4003** (10 µM) for 16 h does not effect YAP/TAZ subcellular localization in LG-derived HTB-2 cells, consistent with the very low levels of NUAK2 expression and predominantly cytoplasmic localization of YAP/TAZ in these cells. Scale bars, 25 µm. **(c)** Blocking NUAK2 expression using siRNAs inhibits the growth of HG bladder cancer cell lines as measured by SRB assay. Data is plotted as the mean +/- SD, n = 3 (TCCSUP) or +/- the range for a representative experiment (T24). NUAK2 knockdown efficiency (right), is plotted as mean +/- range of a representative experiment.

Figure 2a

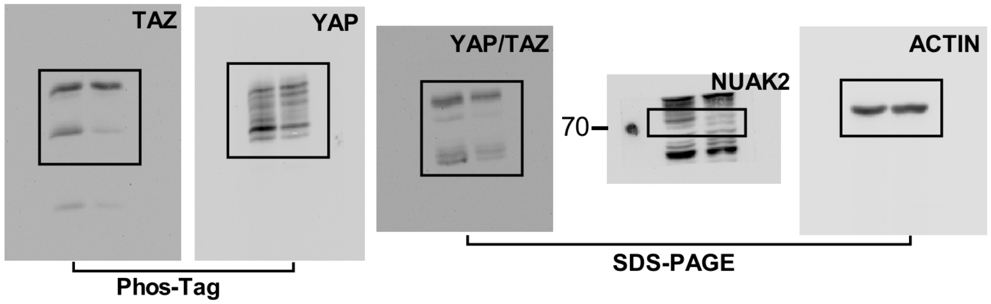

Figure 2c

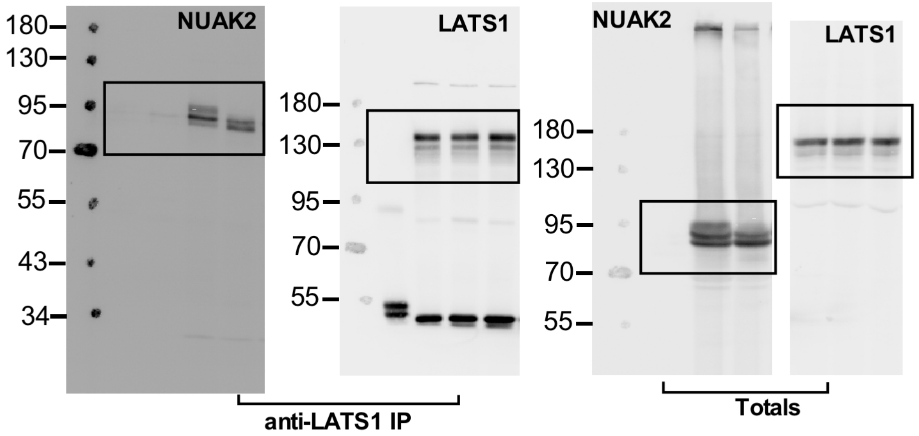

Figure 2f

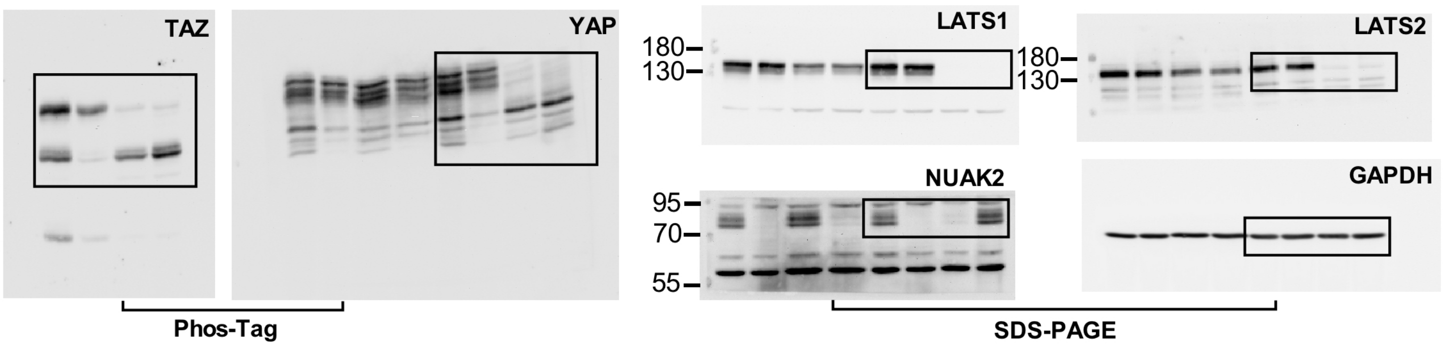

Figure 3a

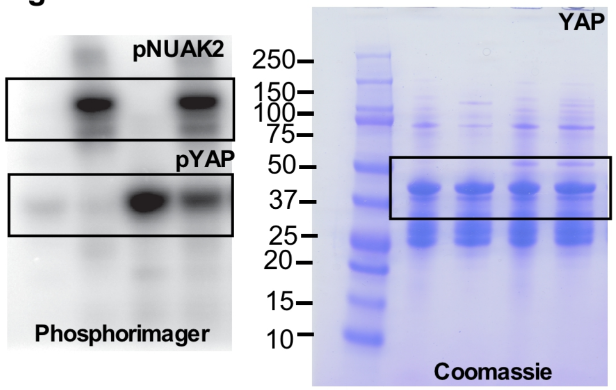

Figure 3c

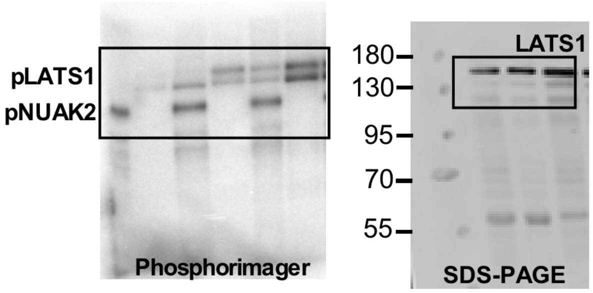

Figure 3d

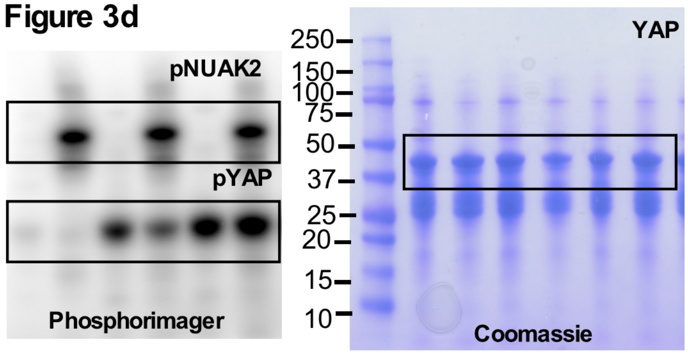

Supplementary Figure 11. Full size immunoblots from Figure 2 and 3.

Figure 4a

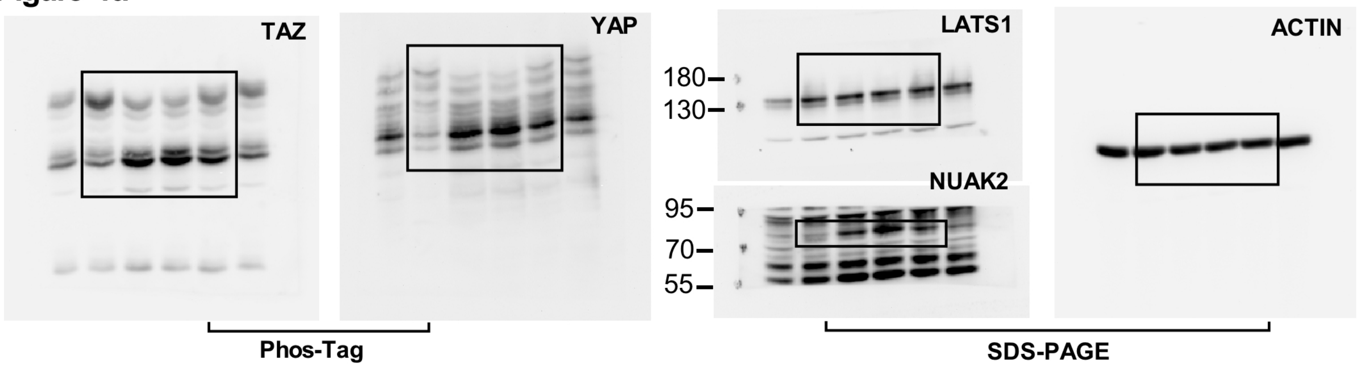

Figure 4e

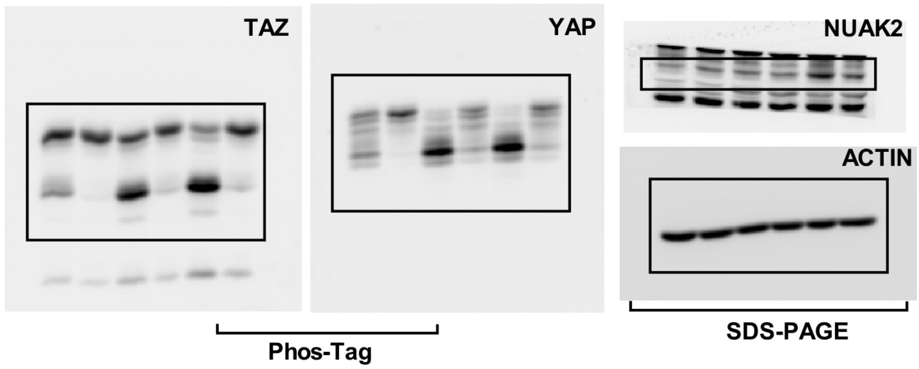

Figure 4g

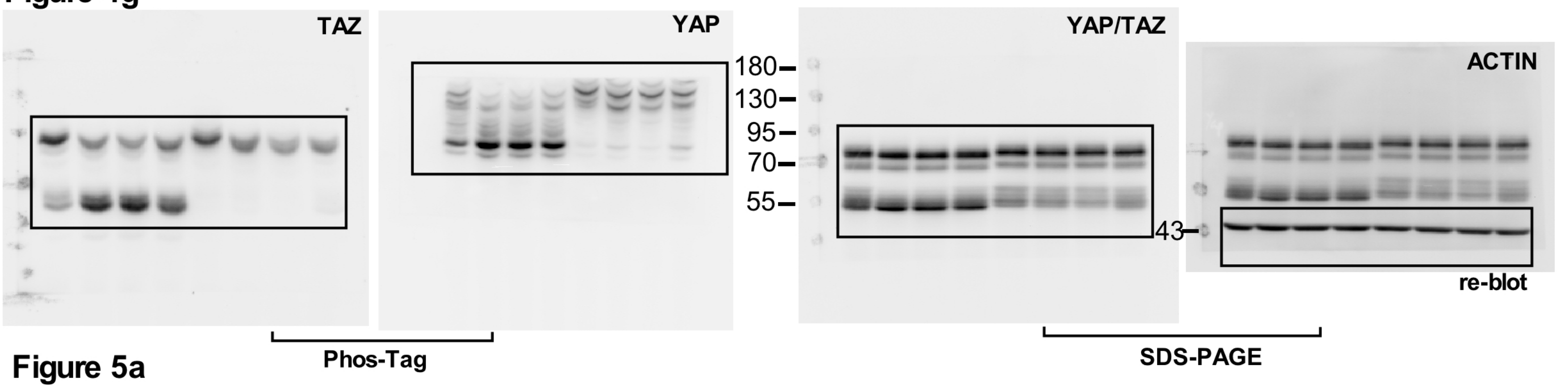

Figure 5a

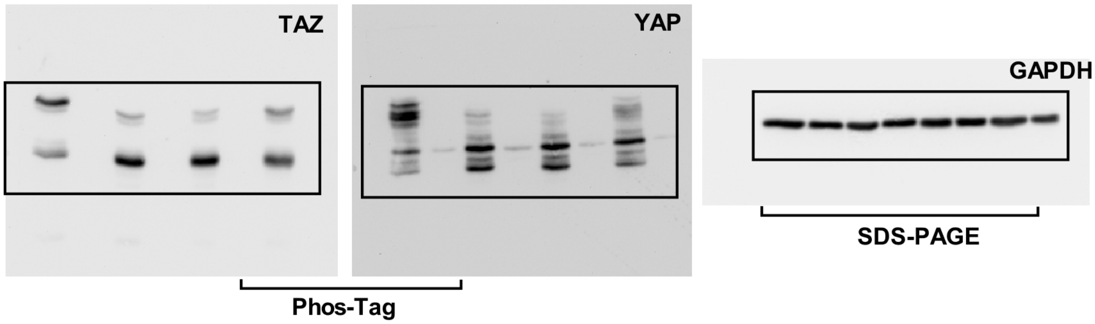

Figure 6g

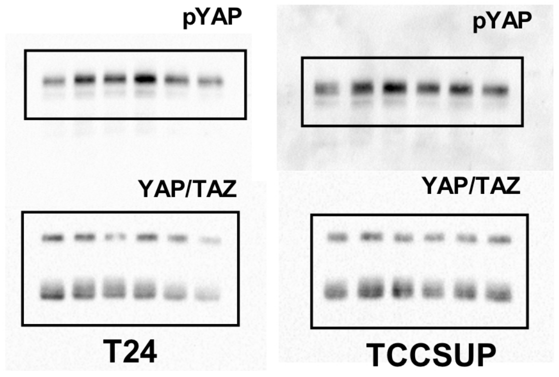

Figure 7i

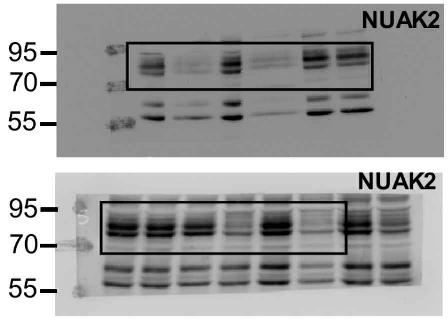

Supplementary Figure 12. Full size immunoblots from Figure 4-7.

Supplementary Table 1. Co-expression of NUAK2 with YAP signature genes in bladder cancer samples

Gene Symbol	Correlation with NUAK2	Gene Symbol	Correlation with NUAK2	Gene Symbol	Correlation with NUAK2
AMOTL2	0.79	DUSP1	0.52	CDKN2C	0.25
CYR61	0.73	ASAP1	0.51	COL4A3	0.23
GADD45B	0.7	THBS1	0.51	SLIT2	0.21
ECT2	0.7	TK1	0.48	GGH	0.2
SERPINE1	0.7	LHFP	0.46	CRIM1	0.18
FLNA	0.69	TOP2A	0.45	NDRG1	0.14
AXL	0.67	PDLIM2	0.44	ITGB5	0.14
GAS6	0.67	TGM2	0.44	STMN1	0.14
ITGB2	0.66	BIRC5	0.43	AGFG2	0.11
TGFB2	0.65	HEXB	0.43	MDFIC	0.09
GLS	0.65	FSTL1	0.42	BICC1	0
SGK1	0.63	DDAH1	0.41	TSPAN3	-0.01
FGF2	0.61	PMP22	0.4	TNNT2	-0.01
CDC20	0.59	FSCN1	0.4	DUT	-0.04
HMMR	0.58	ANKRD1	0.31	PHGDH	-0.05
SCHIP1	0.58	SDPR	0.28	EMP2	-0.15
CTGF	0.55	ETV5	0.28	DLC1	-0.26
SHCBP1	0.53	DAB2	0.27	SH2D4A	-0.38
CENPF	0.53	TNS1	0.25		

Supplementary Table 2

**List of siRNAs**	
**Target gene**	**Dharmacon Catalog #**
Non-targeting	D-001810-03
NUAK2	D-005374-01,05,06,07
NUAK1	D-004931-01,02,03,04
LATS1	D-004632-01,02,03,04
LATS2	D-003865-01,02,03,04
TAZ	D-016083-01,02,03,04
YAP	D-012200-01,02,03,04
NUAK2 (Extended Data Fig. 1c)	D-005374-01,05, pool (D-005374-1,05,06,07) and J-005374-09,08, pool(TP) (J-005374-09,08,10,11).

**Sequence of qPCR Primers**		
**Target**	**Forward**	**Reverse**
HPRT1	ATGGACAGGACTGAACGTCTTGCT	TTGAGCACACAGAGGGCTACAATG
NUAK2	AAGCTGGAGAACATCCTCTTG	GGAACTTGCCTTGATGGTAGA
NUAK2 (KO)	GCTGATCAAGTCGCCCAA	GGGTCTCCAGGAACTCGTA
NUAK1	CAAATCAGCAGCGGAGAGTA	CATCAGCATCCACCGTATGA
ANKRD1	AGTAGAGGAACTGGTCACTGG	TGGGCTAGAAGTGTCTTCAGAT
CTGF	AGGAGTGGGTGTGTGGACGA	CCAGGCAGTTGGCTCTAATC
LATS1	CTCTGCACTGGCTTCAGATG	TCCGCTCTAATGGCTTCAGT
LATS2	ACATTCACTGGTGGGGACTC	GTGGGAGTAGGTGCCAAAAA
YAP1	TGCGTAGCCAGTTACCA	GGTGCCACTGTTAAGGA
TAZ	GTATCCCAGCCAAATCTCGTGATG	CAGCGCATTGGGCATACTCATG
FOSL1	CAGGCGGAGACTGACAAACTG	TCCTTCCGGGATTTTGCAGAT
**Sequence of ChiP-qPCR Primers**		
**Target**	**Forward**	**Reverse**
NUAK2	TCATCTGGGTTCTTCACATTCC	CTCACCTGCCTGGATGC
ANKRD1	AAAAAGGGCAGTGATGTGGTG	GAAGAGGGAGGGGAGGACAA
