# Supplementary material for: A feed forward loop enforces YAP/TAZ signaling during tumorigenesis
Source: Nat Commun. 2018 Aug 29;9:3510. doi: 10.1038/s41467-018-05939-2 (PMC6115388; doi:10.1038/s41467-018-05939-2)
Supplement: Supplementary file 2 — Description of Additional Supplementary Files [file 41467_2018_5939_MOESM2_ESM.pdf]

Description of Additional Supplementary Files

File Name: Supplementary Movie 1
Description: Time-lapse imaging of Clover-YAP WT in MDA-MB231 cells. Localization of Clover-YAP WT was monitored before and after addition of WZ4003. Images were captured every 10 min for 2 hours. Note that the nuclear signal is gradually lost after addition of WZ4003. The scale and timestamp are indicated in the movie.

File Name: Supplementary Movie 2
Description: Time-lapse imaging of Clover-YAP 5SA in MDA-MB231 cells. Localization of Clover-YAP 5SA was monitored before and after addition of WZ4003. Images were captured every 10 min for 2 hours. Note that the nuclear signal of CloverYAP 5SA is not affected by WZ4003 treatment. The scale and timestamp are indicated in the movie.
